# Phylogenetic and genomic analyses of the ribosomal oxygenases Riox1 (No66) and Riox2 (Mina53) provide new insights into their evolution

**DOI:** 10.1186/s12862-018-1215-0

**Published:** 2018-06-19

**Authors:** Katharina E. Bräuer, Kevin Brockers, Jasmin Moneer, Annette Feuchtinger, Evi Wollscheid-Lengeling, Andreas Lengeling, Alexander Wolf

**Affiliations:** 1 Institute of Molecular Toxicology and Pharmacology, Helmholtz Zentrum München-German Research Center for Environmental Health, Ingolstädter Landstrasse 1, 85764 Neuherberg, Germany; 2 Department of Biology II, Ludwig Maximillians University, Munich, Großhaderner Strasse 2, 82152 Planegg-, Martinsried, Germany; 3 Research Unit Analytical Pathology, Helmholtz Zentrum München, German Research Center for Environmental Health GmbH, Ingolstädter Landstr. 1, 85764 Neuherberg, Germany; 4 The Roslin Institute and Royal (Dick) School of Veterinary Studies, University of Edinburgh, Edinburgh, UK; 5 Present address: Max-Planck-Society, Administrative Headquarters, Hofgartenstr. 8, 80539 Munich, Germany

**Keywords:** Ribosome, Ribosomal oxygenases, Fe(II) and 2-oxoglutarate dependent oxygenases, Hydroxylation, JmjC, Jumonji, Mina53, NO66, Single exon genes, Intronless retroposed gene copies

## Abstract

**Background:**

Translation of specific mRNAs can be highly regulated in different cells, tissues or under pathological conditions. Ribosome heterogeneity can originate from variable expression or post-translational modifications of ribosomal proteins. The ribosomal oxygenases RIOX1 (NO66) and RIOX2 (MINA53) modify ribosomal proteins by histidine hydroxylation. A similar mechanism is present in prokaryotes. Thus, ribosome hydroxylation may be a well-conserved regulatory mechanism with implications in disease and development. However, little is known about the evolutionary history of *Riox1* and *Riox2* genes and their encoded proteins across eukaryotic taxa.

**Results:**

In this study, we have analysed *Riox1* and *Riox2* orthologous genes from 49 metazoen species and have constructed phylogenomic trees for both genes. Our genomic and phylogenetic analyses revealed that Arthropoda, Annelida, Nematoda and Mollusca lack the *Riox2* gene, although in the early phylum Cnidaria both genes, *Riox1* and *Riox2,* are present and expressed. *Riox1* is an intronless single-exon-gene in several species, including humans. In contrast to *Riox2*, *Riox1* is ubiquitously present throughout the animal kingdom suggesting that *Riox1* is the phylogenetically older gene from which *Riox2* has evolved. Both proteins have maintained a unique protein architecture with conservation of active sites within the JmjC domains, a dimerization domain, and a winged-helix domain. In addition, Riox1 proteins possess a unique *N*-terminal extension domain. Immunofluorescence analyses in Hela cells and in *Hydra vulgaris* identified a nucleolar localisation signal within the extended *N*-terminal domain of human RIOX1 and an altered subnuclear localisation for the Hydra Riox2.

**Conclusions:**

Conserved active site residues and uniform protein domain architecture suggest a consistent enzymatic activity within the Riox orthologs throughout evolution. However, differences in genomic architecture, like single exon genes and alterations in subnuclear localisation, as described for *Hydra*, point towards adaption mechanisms that may correlate with taxa- or species-specific requirements. The diversification of *Riox1*/*Riox2* gene structures throughout evolution suggest that functional requirements in expression of protein isoforms and/or subcellular localisation of proteins may have evolved by adaptation to lifestyle.

**Electronic supplementary material:**

The online version of this article (10.1186/s12862-018-1215-0) contains supplementary material, which is available to authorized users.

## Background

**Fig. 1:**
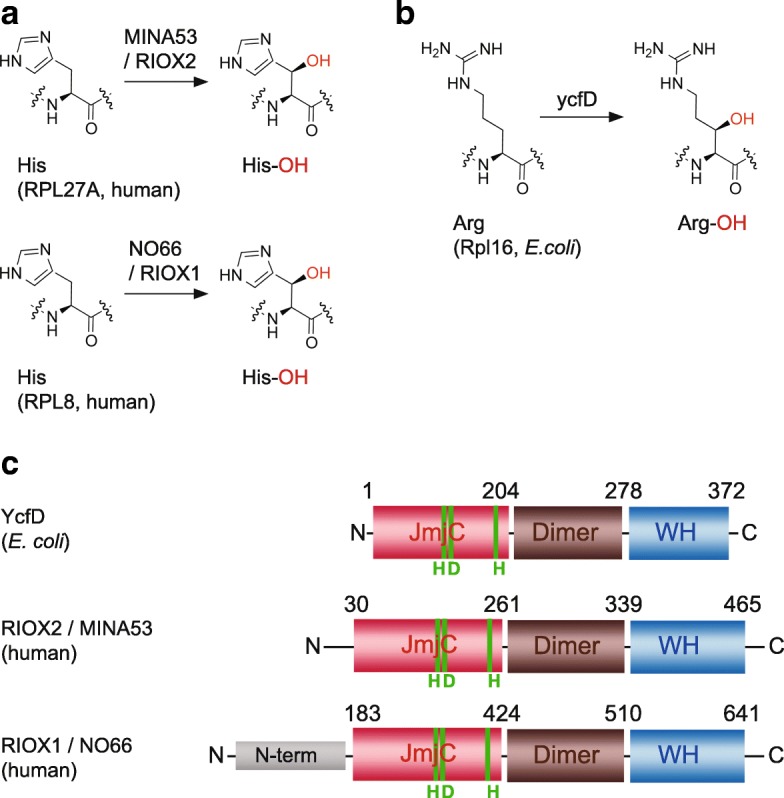
The ribosomal oxygenases (ROXs) are a subgroup of Fe(II) and 2OG-dependent oxygenases that modify the ribosome and are present in pro- and eukaryotes. **a** The human ROXs RIOX2/MINA53 and RIOX1/NO66 hydroxylate histidine residues in the ribosomal proteins RPL27A and RPL8, respectively, whereas (**b**) the *E.coli* ycfD protein hydroxlyates an arginine in Rpl16 [6]. Protein sequence and crystal structure analyses confirmed a similar protein-domain architecture for the three proteins [16]. **c** They consist of a JmjC-domain (red), a dimerization domain (brown) for homo-oligomerization and a winged-helix (WH) domain (blue). The aa triad HxD…H that coordinates the iron and is essential for catalytic activity is indicated in green (**c**)

The central dogma of molecular biology has described gene expression as a straightforward process in which a gene is transcribed into mRNA followed by translation into a protein [1]. In recent years, a multitude of regulatory processes of gene expression have been identified on transcriptional and posttranscriptional levels. The mRNA itself can be subjected to regulatory events such as alternative splicing or RNA modifications [2, 3], but also mRNA translation rates or translation selectivity of individual mRNA molecules can vary [4]. Initially the ribosome was thought to be a rather unaltered ribonucleoprotein particle responsible for translation of any incoming mRNA into a polypeptide. However, recent research has shown that ribosomes are highly specific translational machineries that underlie complex regulatory mechanisms in order to meet physiological requirements in different cell types or throughout development [4]. ‘Specialized’ ribosomes can be generated by various mechanisms, including changes in ribosomal protein composition or posttranslational modifications, like methylation or phosphorylation [5]. Very recently, ribosomal oxygenases (ROXs) have been identified that modify ribosomal proteins by hydroxylation of amino acids [6–10]. The ROXs are a subgroup of the enzyme superfamily of Fe(II) and 2-oxoglutarate (2OG) dependent oxygenases (2OG oxygenases). All known human 2OG oxygenases catalyse transfer of molecular oxygen to a prime substrate, which can be either amino acids or nucleotides [11]. Due to the manifold set of their substrates, 2OG oxygenases have been discovered to have central roles in many cellular processes including epigenetic regulation of gene expression, control of transcriptional initiation, and regulation of alternative splicing [12–15]. The ROXs, which include the nucleolar protein NO66 (official new nomenclature symbol RIOX1) and the MYC-induced nuclear antigen MINA53 (new nomenclature symbol RIOX2), directly modify ribosomal proteins [6, 8, 9]. RIOX1 has been demonstrated to hydroxylate histidine 216 (H216) in the ribosomal protein RPL8 (uL2), whereas RIOX2 hydroxylates H39 in RPL27a (uL15) [6] (Fig. 1a). 2OG-oxygenase-catalysed ribosome hydroxylation is conserved from bacteria to humans. The Riox1/Riox2 counterpart in prokaryotes, ycfD, hydroxylates arginine (R) 81 in Rpl16 [6] (Fig. 1b). In addition, RIOX1 and RIOX2 share similarities in substrate binding with ycfD and exhibit similar conserved protein domains [16]. Crystal structure analyses on recombinant enzymes revealed an *N*-terminal Jumonji C (JmjC) domain, which harbours the active site and the iron-coordinating residues, characteristic for all known 2OG oxygenases. A central dimerization-domain is responsible for homo-oligomerization in-vitro and the *C*-terminus contains a winged-helix (WH) domain [16] (Fig. 1c). Both, human RIOX1 and RIOX2 have been described to be involved in cancer cell growth. *RIOX2* is a myc-target gene [17]. Its expression is upregulated in several cancers, including lung and breast cancer, and knockdown of RIOX2 in A549 cells inhibited cell proliferation [18, 19]. Elevated RIOX2 expression has been described in non-small cell lung cancers [20] and was reported to be associated with invasive colorectal cancer [21]. In addition, RIOX2 regulates immune responses as a transcriptional co-repressor of the *interleukin-4* (*Il4*) encoding gene [22]. It has been described to polarize T helper 2 (Th2) cell responses in atopic pulmonary inflammation and to have a role in parasitic worm expulsion [23]. Riox1 is involved in osteoblast differentiation [24] and variations in its expression level have been reported to regulate skeletal growth and bone formation in mice [25, 26].

Here we provide the first comprehensive sequence analyses of the ribosomal oxygenases Riox1 and Riox2 in different eukaryotic species. We compared the domain architecture of both proteins and their exon-intron gene structures across a wide range of metazoan species. In addition we used immunofluorescence approaches to investigate expression in human cells and in the Cnidaria *Hydra vulgaris*.

## Results

### Evolutionary sequence and protein domain architecture conservation of the ribosomal oxygenases Riox1 and Riox2

**Fig. 2:**
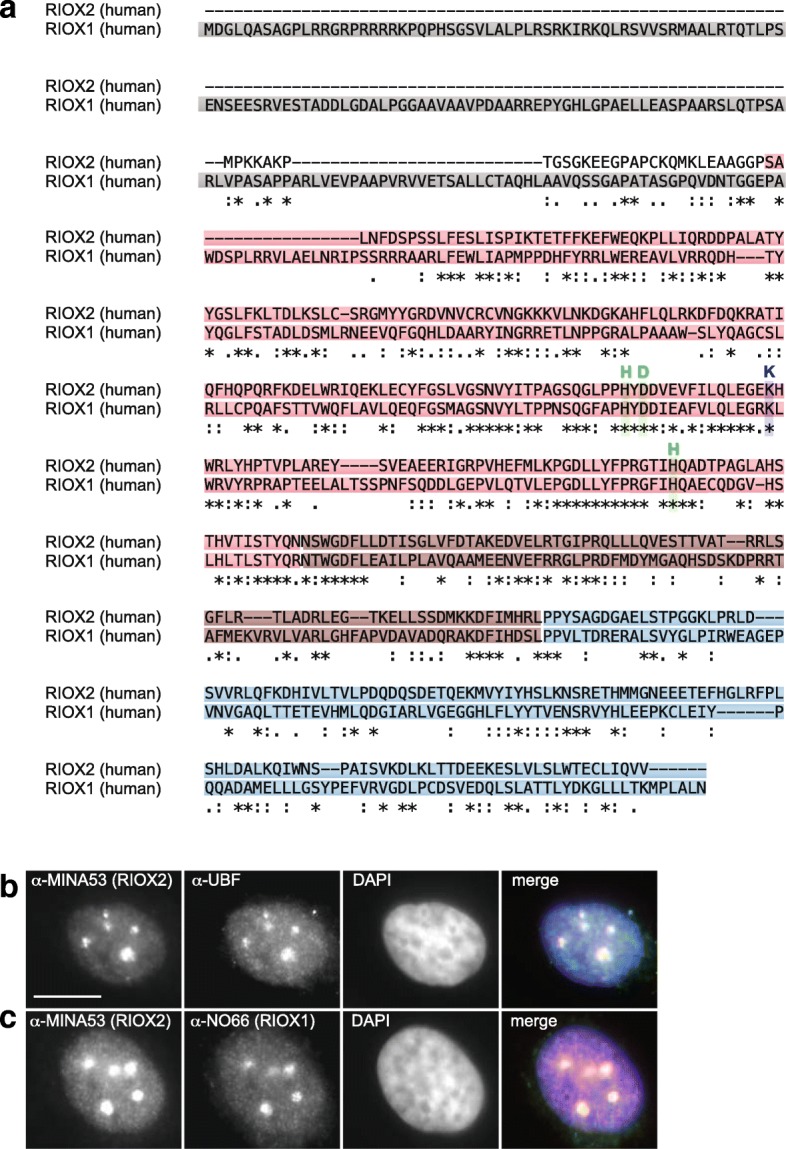
Clustal omega sequence alignment [35] of human RIOX2 and RIOX1 aa sequences. **a** Protein domains are indicated in red (JmjC), brown (dimerization) and blue (WH). The RIOX1-specific *N*-terminal extension is marked in grey. The Fe(II) and 2OG-binding residues are highlighted in green or purple respectively. Both, human RIOX2 and RIOX1 localise to nucleoli. Immunofluorescent staining of endogenous RIOX2 (α-Mina53) in HeLa cells showed co-localisation with (**b**) the nucleolar marker protein upstream-binding factor 1 (α-UBF) and (**c**) with RIOX1 (α-NO66). Scalebar: 10 μm

A sequence alignment of human RIOX2 (MINA53, 465 amino acids (aa)) and human RIOX1 (NO66, 641 aa) proteins revealed 23.2% identical and 16.4% similar aa (Fig. 2a). Both proteins exhibit the same protein domain structure, including a JmjC domain, a dimerization domain and a WH domain [16]. The RIOX1 protein has an *N*-terminal extension of 184 aa, which is absent in the RIOX2 protein (Fig. 2a). RIOX1 and RIOX2 are nuclear proteins with a strong accumulation in nucleoli [27, 28] (Fig. 2b,c). To identify *Riox1* and *Riox2* orthologs in other eukaryotic species we searched the genomes of the model organisms *Mus musculus*, *Gallus gallus*, *Danio rerio*, *Caenorhabditis elegans* and *Drosophila melanogaster*. An Ensembl genome browser search with the human RIOX1 protein sequence (Ensembl: ENSG00000170468; Uniprot: Q9H6W3) revealed orthologous proteins in *M. musculus*, *G. gallus*, *D. rerio*, *C. elegans* and *D. melanogaster*. The *M. musculus* Riox1 protein sequence (Ensembl: ENSMUSG00000046791; Uniprot: Q9JJF3) showed 75.2% identity to the human sequence, with most sequence variation in the *N*-terminal extension domain (Additional file 1: Figure S1). The *G. gallus* Riox1 protein (Ensembl: ENSGALG00000020454; Uniprot: Q5ZMM1) showed 49.3% identity (Additional file 2: Figure S2), the *D. rerio* Riox1 43% (Ensembl: ENSDARG00000067838; Uniprot: A3KP59) (Additional file 3: Figure S3), the *D. melanogaster* Riox1 33.1% (Ensembl: FBgn0266570; Uniprot: E2QD64) (Additional file 4: Figure S4) and the *C. elegans* Riox1 protein (Ensembl: WBGene00020902; Uniprot: O01658) 28.7% aa sequence identity (Additional file 5: Figure S5).

A search for *Riox2* sequences in the same set of organisms revealed no Riox2 orthologs in *C. elegans* and *D. melanogaster*. Searches with human RIOX2 (Ensembl: ENSG00000170854; Uniprot: Q8IUF8) or *D. rerio* Riox2 (Ensembl: ENSDARG00000036359; Uniprot: F1R7K2) sequence respectively revealed no homologous sequences, but detected the above described Riox1 sequences for *C. elegans* and *D. melanogaster* as the sequences with highest homology.

Individual alignments of Riox2 sequences with the human RIOX2 showed 76.3% identical aa for the *M. musculus* Riox2 sequence (Ensembl: ENSMUSG00000022724; Uniprot: Q8CD15) (Additional file 6: Figure S6), 70.8% for the *G. gallus* Riox2 (Ensembl: ENSGALG00000039302; Uniprot: E1C6P1) (Additional file 7: Figure S7) and 57.2% for the *D. rerio* Riox2 (Ensembl: ENSDARG00000036359; Uniprot: F1R7K2) (Additional file 8: Figure S8).

**Fig. 3:**
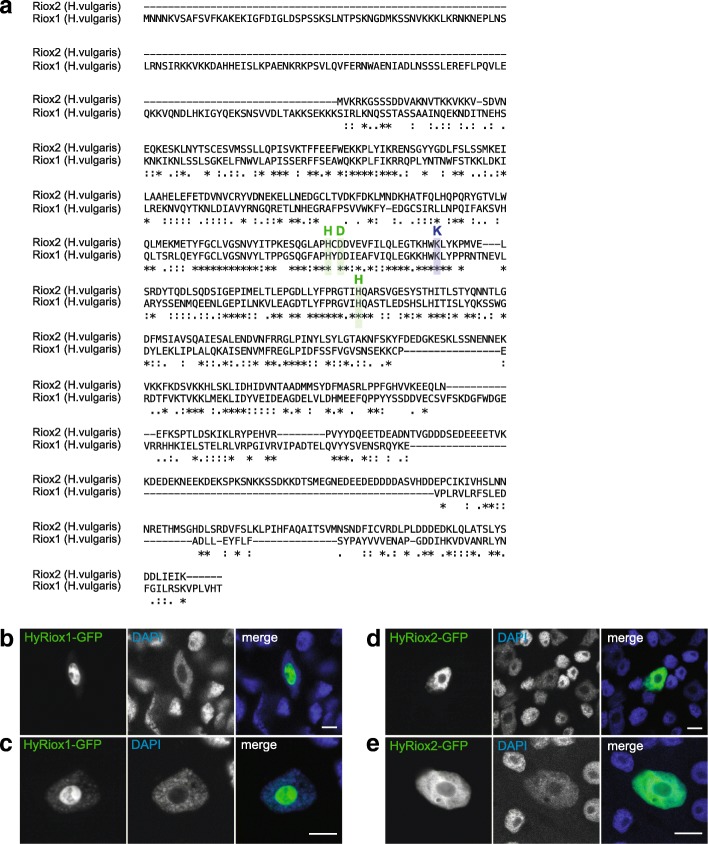
Riox1 and Riox2 expression in Hydra. **a** Alignment of both *Hydra* sequences, with highlighted iron-binding motif (HxD…H) (green) and predicted lysine-residue for 2OG binding (blue). **b**, **c** Expression of GFP-tagged HyRiox1 in Hydra animals displayed nuclear localisation and strong accumulation in nucleoli. **d**, **e** HyRiox2 is also localised in nuclei, but an accumulation in nucleoli is not detectable. DNA-stain: DAPI. Scalebar: 10 μm

Genome sequence searches of additional invertebrate species from the Arthropoda, Annelida, Nematoda, and Mollusca clades also did not identify any putative *Riox2* orthologous genes (results discussed below), but the early metazoan animal *Hydra vulgaris* of the phylum Cnidaria exhibited both, *Riox1* and *Riox2* sequences (Fig. 3). That prompted us to analyse the expression of the hydra orthologs in more detail.

### Characterization and cellular localization of hydra Riox1 and Riox2 proteins

The fresh-water polyp *Hydra* is a pre-bilaterian animal of the phylum Cnidaria [29]. Database searches of the *Hydra vulgaris* genome [30] predicted two sequences encoding for *Hydra Riox2* (HyRiox2) and *Hydra Riox1* (HyRiox1). Amplification with corresponding primers, cloning and subsequent sequencing identified the predicted HyRiox2 (551aa) and HyRiox1 (628aa) protein sequences encoded in *Hydra* cDNAs. An alignment of these *Hydra* protein sequences with the human RIOX1 and RIOX2 proteins revealed 35.0 and 33.7% aa sequence identity, respectively (Additional file 9: Figure S9 and Additional file 10: Figure S10). A sequence comparison of the two identified *Hydra* ROXs showed, that HyRiox2 and HyRiox1 share 19.7% identical aa (Fig. 3a). ROXs proteins are characterized by a double-stranded beta-helix fold (DSBH or JmjC domain) that harbours the active site, including residues for co-factor (Fe(II)) and co-substrate (2OG) binding [11]. Based on the crystal structures of human RIOX1 and RIOX2 proteins [16], both *Hydra* sequences exhibit the characteristic Fe(II) binding motif HxD/E…H (HyRiox2: H178xD180…H240; HyRiox1: H330xD332…H395) and also a conserved 2OG C5-carboxylate-interacting lysine (K) residue (HyMina53: K193; HyNo66: K345) (Fig. 3a; Additional file 9: Figure S9, Additional file 10: Figure S10). Both *Hydra* homologs have also the characteristic ROX protein domain architecture with JmjC, dimerization, and WH domains. In addition, the *Hydra* Riox1 homolog HyRiox1 exhibits an *N*-terminal extension domain proximal to the JmjC domain. Interestingly, the HyRiox2 contains two extended sequence segments in its dimerization domain and one extended sequence stretch in the WH domain. No corresponding sequences of these extensions are detectable in the human RIOX2 protein. The sequence extension in the HyRiox2 WH domain comprises a stretch of 64 additional residues, rich in the charged aa lysine (K), aspartate (D) and glutamate (E) (42 of the 64aa) (Additional file 9: Figure S9).

To analyse the subcellular localization of HyRiox2 and HyRiox1, we expressed GFP-tagged full-length proteins in *Hydra* cells of intact animals after transfection with a particle gun [31]. Confocal imaging of ectopically expressed GFP-tagged HyRiox1 confirmed the nuclear localization of the protein with a prominent accumulation in nucleoli (Fig. 3b,c). Transfection of HyRiox2 constructs revealed also expression in the nucleus; however, in contrast to the human RIOX2, HyRiox2 did not accumulate to nucleoli (Fig. 3d,e).

### Sequence analyses of Riox1 and Riox2 proteins from different species

The ROXs domain topology of homologous Riox1 proteins is similar in all analysed species. The lengths of their JmjC domains (Riox1: 236-242aa), dimerization domains (Riox1: 86-93aa) and WH domains (Riox1: 129-133aa) are comparable in the analysed organisms, whereas the lengths of the *N*-terminal extension varies from 87 in *D. rerio* to 286 aa in *C. elegans* (Additional file 11: Figure S11). An alignment of these Riox1 protein sequences showed strong conservation of the Fe(II) and 2OG-binding residues in the catalytic site of these enzymes (Additional file 11: Figure S11). The sequence homology implies a conserved function of Riox1 across species. Structural analysis of human RIOX1 co-crystallised with an Rpl8 peptide identified interactions of arginine (R) 297, tyrosine (Y) 328 and serine (S) 421 with the substrate peptide [16]. These residues are conserved in all Riox1 proteins across the analysed species (Additional file 11: Figure S11). Similarly, in Riox2 (Additional file 12: Figure S12), four primary amides (asparagine (N) 101, glycine (G) 136, G139, N165) and S257 in human RIOX2 have been identified to interact with its Rpl27a substrate peptide. These residues and the Fe(II) and 2OG-binding sites are all conserved in Riox2 proteins across species throughout evolution (Additional file 12: Figure S12). Alignment of Riox2 sequences from human, mouse, chicken, zebrafish and *Hydra* revealed additional aa stretches in the zebra fish and *Hydra* proteins, which are not present in the other species. The zebra fish Riox2 displayed an additional 30aa stretch between the JmjC and the dimerization domains (Additional file 12: Figure S12). The *Hydra* sequence has an extended dimerization domain and an additional 64aa stretch within its WH domain (Additional file 12: Figure S12).

The aforementioned conserved active site and substrate binding residues in Riox1 and Riox2 prompted us to look into the sequence homology of their known substrates Rpl8 and Rpl27a in corresponding species. Comparing the Rpl8 and Rpl27a sequences of 39 different eukaryotic species available in the Ribosomal Protein Gene Database (http://ribosome.med.miyazaki-u.ac.jp) [32] revealed that a corresponding H residue that may be hydroxylated by Riox1 or Riox2 can be found in each species (data not shown). An alignment of the aa-stretches surrounding the H hydroxylated by human RIOX1 and RIOX2 showed almost identical aa-sequences for Rpl8 and Rpl27a from ten different species (Additional file 13: Figure S13A,B).

### The N-terminal extension domain in Riox1

**Fig. 4:**
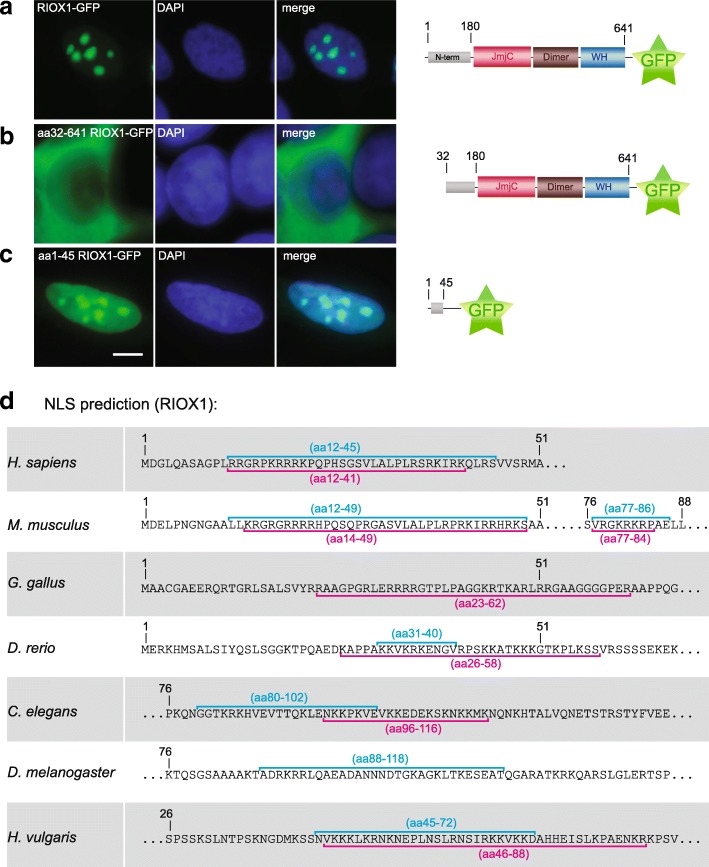
Nuclear localisation of Riox1 and Riox2. The *N*-terminal extension domain of human RIOX1 has been shown to harbour the nuclear localisation signal (NLS) [28]. **a** Expression of GFP-tagged full-length RIOX1 in HeLa cells resulted mainly in nucleolar accumulation. **b** A RIOX1 deletion mutant lacking aa 1–31 localised to the cytoplasm. **c** Fusion of aa 1–45 of human RIOX1 resulted in nuclear GFP localisation with strong accumulation in the nucleoli. DNA stain: DAPI. Scalebar: 5 μm. **d** Predictions of NLS in Riox1 of other species with either NLSmapper (blue) or NLStradamus (pink) identified NLS in the *N*-terminal extension domains of Riox1, whereas for Riox2 no NLS were predicted

Despite the characteristic domain structure of the ROXs, an obvious difference between Riox2 and Riox1 is the *N*-terminal extension present in the Riox1 sequences. This *N*-terminal extension domain has no assigned function yet, but aa 1–31 have been predicted to possess a nuclear localization signal [28]. We further investigated this hypothesis using green fluorescent protein (GFP) fusion reporter experiments. Full-length human RIOX1 tagged with GFP localized predominantly in nucleoli of transfected HeLa cells (Fig. 4a). In contrast, a GFP-tagged deletion mutant lacking aa 1–31 displayed an exclusive cytoplasmatic localisation (Fig. 4b). When we fused the N-terminus of human RIOX1 (aa 1–45) to GFP, the fusion protein localised to the nucleus and accumulated in nucleoli as well (Fig. 4c). We used two different bioinformatic tools (NLSmapper & NLStradamus) [33, 34] to predict nuclear localization signals (NLS) in Riox1 of several species. In each species, the NLS motive was identified with NLSmapper and NLStradamus tools within the *N*-terminal extension of the analysed Riox1 proteins (Fig. 4d). In contrast, the mechanism of how Riox2 is directed into the nucleus and/or nucleolus is to our knowledge not known yet. Both NLS prediction tools did not identify corresponding NLSs in all analysed Riox2 protein sequences (data not shown).

### Comparative genomic analysis of *Riox1* and *Riox2* across the animal kingdom

**Fig. 5:**
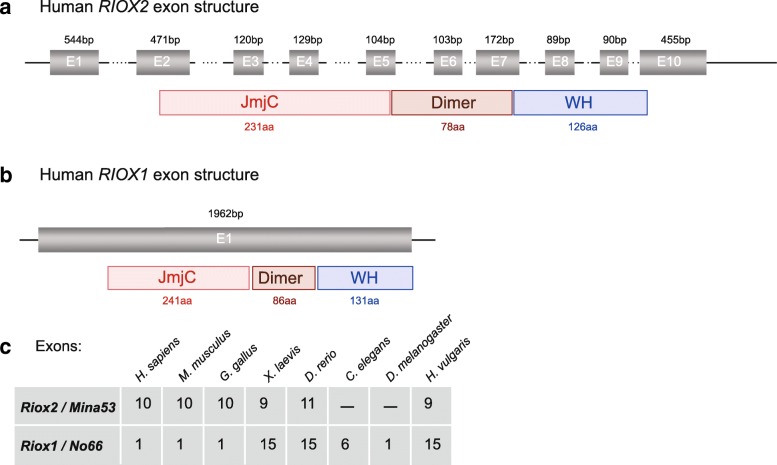
Riox1 and Riox2 gene structures. **a** The human *RIOX2* gene exhibits ten exons distributed on chromosome 3. The exon lengths are indicated. The RIOX2 protein domains (JmjC, dimerization, WH) were mapped on the gene. **b** The human *RIOX1* gene is a single exon gene of 1962 bases on chromosome 14. The RIOX1 protein domains (JmjC, dimerization, WH) were mapped on the gene. **c** Analysis of the genomic structures of *Riox2* and *Riox1* genes in *H. sapiens*, *M. musculus*, *G. gallus*, *X. laevis*, *D. rerio*, *C. elegans*, *D. melanogaster* and *H. vulgaris* with the number of exons are given in the Table. *C. elegans* and *D. melanogaster* lack a *Riox1* gene. The *Riox2* genes of human (Hs), mouse (Mm) and chicken (Gg) exhibit one non-coding exon (5’) and nine coding exons

Analyses of the human *RIOX1* and *RIOX2* genes using the Ensembl genome browser portal revealed 10 exons for human *RIOX2.* The gene encodes 9 different transcripts and comprises 9 coding exons and alternatively used non-coding 5’-UTR and 3-UTR exons. The largest transcript, RIOX2–001 (ENST00000333396.11), encodes a protein of 465 aa and encompasses a locus of 30,639 base pairs (bp) on human chromosome 3 (Fig. 5a). Surprisingly, the human *RIOX1* gene encodes one transcript RIOX1–003 (ENST00000304061.7) and is an intronless gene, spanning 2428 bp with 5′- and 3’-UTRs on chromosome 14 (Fig. 5b). Mapping of the characteristic ROX protein domains (JmjC, dimerization, WH) to the exon-intron gene structure of *RIOX2* revealed that the JmjC domain is encoded by exons 2–4 and part of exon 5. *RIOX2* exons 5, 6 and part of exon 7 encode the dimerization domain, whereas the WH domain is encoded by exons 7, 8, 9 and part of 10 (Fig. 5a). When we compared genomic structures of *Riox1* and *Riox2* orthologues throughout evolution we found that *Riox2* comprises ten (nine coding and one non-coding 3’-UTR) exons in human, mouse and chicken or 9 coding exons in *Xenopus*, 11 exons in zebra fish and 9 exons in *Hydra* (Fig. 5c).

**Fig. 6:**
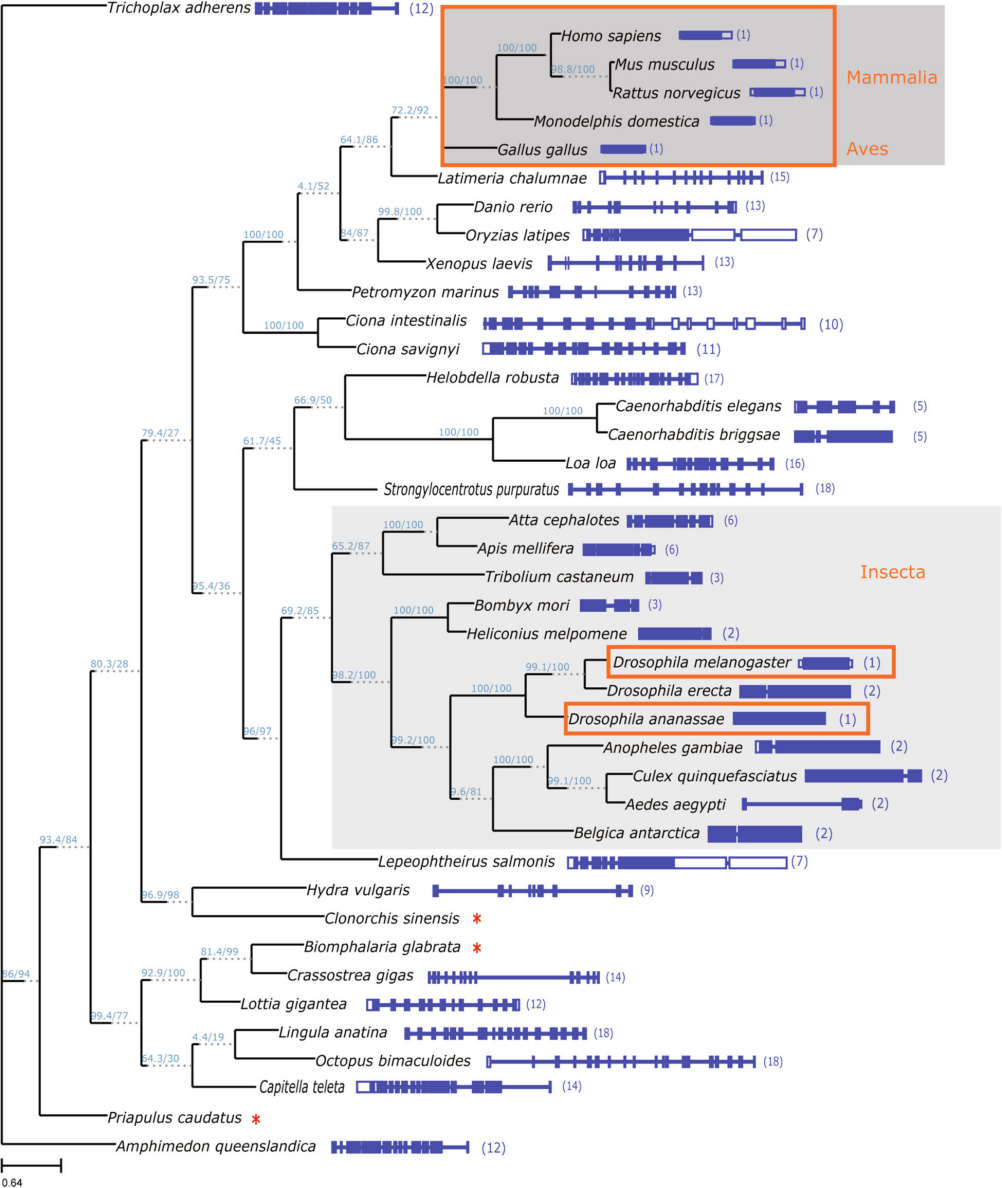
Phylogenetic tree of Riox1 (No66) orthologues proteins and exon-intron architecture of the corresponding protein encoding genes. The tree was inferred through a maximum-likelihood analysis of 41 representative species (IQ-TREE). The tree shown is a consensus tree with SH-like aLRT and ultrafast bootstrap (UFboot) values (numbers in parentheses SH-aLRT support (%)/ultrafast bootstrap support (%)) given as branch support values. Good branch support is confirmed with SH-aLRT > = 80% and UFboot > = 95%. The Tree is unrooted although the outgroup taxon ‘Trichoplax’ is drawn at root. The scale bar indicates 0.64 substitutions per site. Blue boxes and lines on the right show the gene architectures of the corresponding genes with exons and introns, respectively. Filled boxes represent protein-coding exons, empty boxes represent non-coding 5′- and 3’-UTR exons. Numbers in parentheses indicate total number of exons. Red rectangles encircle single exon, intronless genes present in three different taxa (Mammalia, Aves, and Insecta, grey background shading). Stars indicate species for which completed (non-fragmented) gene architecture annotations are yet not available

To gain additional insights into the evolution of the *Riox1* and *Riox2* genes we included additional metazoan species that represent key taxa from the animal kingdom and constructed phylogenetic trees for both genes. Protein sequences encoded by *Riox1* and *Riox2* orthologues were identified in the Ensembl and EnsemblMetazoa genomic resources using the human RIOX1 and RIOX2 protein reference sequences in Protein BLAST (blastp) search routines or the Ensembl evidence-based annotation of orthologues from pre-existing whole-genome pairwise alignments which are available on both Ensembl portals. Obtained protein sequences were validated as being true orthologs using the following criteria: (i) Independent blastp searches with the *Homo sapiens* RIOX1/RIOX2 and *Hydra vulgaris* Riox1/Riox2 reference sequences mapped to a single locus in the respective genome with an E-value < 1 × 10^− 40^; (ii) Multiple blastp sequence hits (> 2) were obtained with the human and *Hydra* query sequence in the same locus; and (iii) The extracted full-length protein sequence followed the characteristic domain structure of ROX proteins. As an additional criterion for correct assignment of *Riox1* orthologs, we also included the presence of the above described *N*-terminal extension domain in the candidate sequence. All Riox1 ortholog sequences downloaded from the Ensembl portals matched these criteria with the exception of the *Trichoplax adhaerens* sequence, which contained a JmjC domain but had neither a dimerization nor a WH domain. Thus, we could not classify this *Trichoplax adhaerens* JmjC protein as an *Riox1* or *Riox2* orthologous gene, however, we included the sequence in the multiple sequence alignments to root the generated phylogenetic trees to a basic metazoan species for which we had sufficient high quality sequence information available on the EnsemblMetazoa portal. The protein phylogenetic analysis of *Riox1* orthologs together with the architectures of their genes as annotated in Ensembl reveals that intronless *RIOX1/Riox1* genes have evolved independently at least two times in the animal kingdom. Intronless *RIOX1/Riox1* genes are present in the mammalian lineage (humans, mice, and rats), in the marsupial opossum *Monodelphis domestica*, and in chicken as the representative species of the avian lineage (Fig. 6). Fish, amphibians and other chordates all possess multi-exon genes with up to 15 exons (*Latimeria chalumnae*) and differently sized and structured non-coding 5′- and 3’-UTR exons (Fig. 6). Within the clade Insecta at least another evolution of an intronless *Riox1* gene must have taken place. We identified intronless loci in *Drosophila melanogaster, Drosophila ananassae,* and 7 other *Drosophila* species that encoded intact, full-length open reading frames (ORFs) for Riox1 proteins within a size range of 653 to 907 aa (Fig. 6, and data not shown). Importantly, these intronless *Riox1* genes are not clustered within the Insecta clade. Other *Drosophila* species like *D. erecta*, *D. mojavensis*, and *D. sechellia* have *Riox1* genes that consist of 2 exons and phylogenetically related flies such as *Anopheles gambiae*, *Culex quinquefasciatus*, *Aedes aegypti*, and *Belgica antarctica* also possess *Riox1* genes with two exons. Other representative species of the Insecta clade such as *Tribolium castaneum* and *Atta cephalotes* have multi-exon *Riox1* genes, however, in contrast to the majority of other analysed metazoan species the number of exons in these insects does not exceed a total number of 6.

**Fig. 7:**
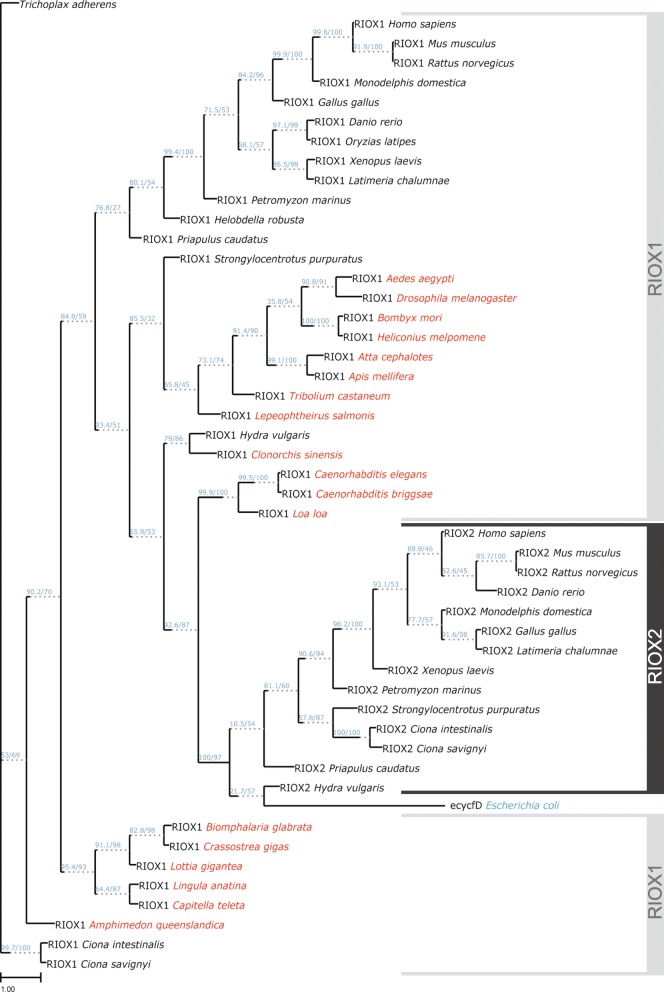
Phylogenetic relationship of Riox1 and Riox2 JmjC domain sequences in Metazoa. Riox1- and Riox2-JmjC domain sequences from species used in this study were extracted from full-length protein sequences, aligned using ClustalW and maximum-likelihood analysis used for tree construction (IQ-TREE). The tree shown is a consensus tree with SH-like aLRT and ultrafast bootstrap (UFboot) values (numbers in parentheses SH-aLRT support (%)/ultrafast bootstrap support (%)) given as branch support values. Good branch support is confirmed with SH-aLRT > = 80% and UFboot > = 95%. The Tree is unrooted although the outgroup taxon ‘Trichoplax’ is drawn at root. The scale bar indicates 1.00 substitutions per site. The JmjC-domain of the *E. coli* ycfD ribosomal oxygenase (ecycfD) was included in the alignment to analyse its phylogenetic relationship to metazoan Riox1 and Riox2 proteins (indicated in red). Riox2-JmjC domain branch is highlighted with grey background shading to show its separate branch node relationship to Riox1-JmjC domains. Note, Riox2 is also present in *Hydra vulgaris* and *Priapulus caudatus* which both possess *Riox1* and *Riox2* orthologous genes as invertebrates. Species with a *Riox1* gene, but which lack a *Riox2* gene are highlighted in red

Protein sequence alignments [35] of Riox1 and Riox2 JmjC domains obtained from 49 species and the *Escherichia coli* (*E.coli*) ROX protein ycfD and construction of phylogenetic trees using maximum likelihood and bootstrap analysis (data not shown) revealed congruent tree topology. Our phylogenetic study shows a strong node support for a Riox2-JmjC domain branch clearly separating this from Riox1-JmjC domains (Fig. 7). Within the Riox2 branch only closely related taxa (e.g. *Ciona*) or taxonomic higher ranks (e.g. Chordata) possess high node support making an assumption about the diversification process and possible functions of Riox2 in different taxa difficult. The strong support for the branching node Riox2 *Hydra vulgaris*/ecycfD *E. coli* to the remaining Riox2-possessing species suggests an early invention of Riox2. Interestingly, Riox2 showed the closest sequence relatedness to the nematode Riox1 analogs, demonstrating that *Riox2* has evolved in the chordata lineage from an ancestral *Riox1* gene (Fig. 7). Again strong support for Riox1 Jmjc domains relatedness is given for closely related taxa (e.g. *D. rerio* + *O. latipes*, *B. mori* + *H. melpomene*) or higher taxa ranks (e.g. Mammalia, Chordata, Mollusca). Weak node support (e.g. *H. robusta* + Chordata, *S. purpuratus* + *L. salmonis*/Insecta) indicates that additional research is needed to fully understand the evolution of this gene and its functional diversification.

## Discussion

Although both proteins, Riox1 and Riox2 are present in *Hydra vulgaris*, our phylogenetic analyses revealed the absence of Riox2 in all other investigated invertebrate lineages with the exception of the small phylum Priapulida represented by the marine worm *Priapulus caudatus*. All analysed Chordata and Echinodermata, and Hyperoartia possess a *Riox2* gene. This suggests that at the basis of the Chordata, which are represented by the Tunicata *Ciona intestinalis* and *Ciona savignyi*, Riox2 was first evolved from a common ancestral *Riox* gene. In contrast, *Riox1* is ubiquitously present throughout the animal kingdom. This suggests that *Riox1* is the phylogenetically older gene from which *Riox2* evolved, possibly in two independent events in early Cnidaria (*H. vulgaris*) and Priapulida (*P. caudatus*). The sequence homology of the *H. vulgaris* Riox2 JmjC domain to the JmjC domain of the *E. coli* ycfD protein (Fig. 7) suggests that Riox2 has a ribosomal oxygenase function like its prokaryotic analog [6].

Comparison of the genomic architecture manifested a common exon/intron structure for *Riox2* genes throughout evolution. In all analysed organisms, the *Riox2* gene consists of 9–10 exons. In contrast, *Riox1* genes possess strikingly different gene structures throughout the animal kingdom. All Mammalia and Aves (birds) have intronless *Riox1* genes (Fig. 6). Insecta have *Riox1* genes with few exons (2 to 6) with some species of *Drosophila* possessing also single exon genes (Fig. 6).

This suggests two possible scenarios of gene evolution in the Insecta lineage. One possibility is that the *Drosophila* clade has acquired an intronless *Riox1* gene at the branch point of divergence from other fly species and this intronless gene copy has been maintained in particular *Drosophila* species. Alternatively, a single exon *Riox1* gene was evolved by retroduplication at the branch point of all Insecta and introns were then independently acquired in different fly subclades with the exception of the *Drosophila* clade, in which intronless *Riox1* genes have been maintained in some but not all *Drosophila* species. In the latter scenario, an evolutionary selection pressure must have preserved the maintenance of those intronless genes in particular *Drosophila* species. Reasons for such a preservation of intronless *Riox1* loci throughout evolution are unknown but as discussed below different explanations are possible.

Approximately 9% of all genes in the human genome are intronless [36]. Intronless genes are typically expressed at a lower level compared to intron-containing genes and evolve through retroduplication events in which mRNA transcripts are reverse-transcribed into DNA and inserted into genomes [37–39]. Such retroposition events are an important mechanism for the origination and evolution of new genes from ancestral, parental source genes [38, 40]. New retropositioned genes may eventually evolve new functions through recruitment of promoter and/or enhancer elements from other regions in the genome and may thereby acquire new spatiotemporal expression patterns, which are different from the ancestral source gene [40]. This can then result in novel tissue- or cell-specific functions of retroposed intronless genes [37]. Interestingly, *Riox1* single exon genes are mainly present in taxa with terrestrial lifestyles. Taxa representing animals with predominantly aquatic lifestyles have multiexonic genes with up to 18 exons (Fig. 6). The reason for this divergence in *Riox1* gene architectures is unknown but it may be related to oxygen content and physiological requirements in these diverse environments. Riox proteins are members of the JmjC protein superfamily and as such their enzymatic dioxygenase activity highly depends on the concentration and availability of molecular oxygen [11]. Multiexonic genes and their capability to express multiple Riox1 isoforms with different functions might better suit aquatic organisms, that are dependent on dissolved oxygen for respiraton to adapt to different environments. In contrast, intronless genes have generally a narrower expression pattern and are usually also expressed at lower levels compared to multiexonic genes. With human and mouse *Riox1* genes we have undertaken a preliminary in silico expression analysis with publically available microarray datasets and expression atlases of primary cells and tissues using the network analysis tool BioLayout/Miru [41, 42]. Searches for *Riox1* co-expression networks did not identify defined clusters of co-expressed genes, probably due to the low level and ubiquitous expression pattern of *Riox1* in most analysed cells and tissues (data not shown). However, many intronless genes are known to be associated with stress or immune response induced signalling pathways, which require fast induction of gene and protein expression. Here, the circumvention of complex primary mRNA processing steps such as splicing may insure faster response rates of expression induction. Examples of such intronless genes are members of the heat shock 70 gene family [43], interferon-coding genes [44] and genes encoding G protein-coupled receptors [45]. Future research may identify conditions under which *Riox1* expression is specifically induced and these might be related to stress inducing conditions. This hypothesis is supported by the finding that the closely sequence related paralog of *Riox1* and *Riox2*, *Jmjc-1* of *Caenorhabditis elegans*, is involved in the regulation of an evolutionary conserved stress-response network [46]. In addition, ycfD has been very recently identified in the thermos- and halophilic obligate aerobe *Rhodothermus marinus* (*R. marinus*). The marine *R. marinus* of the bacterial phylum Bacteriodetes grows at temperatures around 65 °C but its ycfD is also able to catalyse Arg hydroxylation of Rpl16 (uL16) [10]. By now ribosomal oxygenases have been identified in bacteria and eukaryotes, though they seem to be not present in archea [16].

Our protein sequence analyses of the ribosomal oxygenases Riox1 and Riox2 in several eukaryotic organisms revealed a conserved protein domain architecture, which initially has been identified by X-ray crystallography in human RIOX2, RIOX1 and the prokaryotic yfcD. However, the lengths of these three domains (JmjC, dimerization, WH) is limited by a range of variation with some exceptions for organisms exhibiting specific extended sequence elements. As genome sequence coverage and qualities of genome assembly still varies among the analysed organisms some of these extended sequence elements might represent annotation mistakes generated by Ensembl’s automatic genebuild pipeline. In the cnidarian *Hydra vulgaris* the Riox2 sequence encodes two extended aa stretches with no corresponding matches in the human sequence. Cloning of the respective *Hydra* Riox2 from cDNA confirmed the occurrence of those sequence elements. Interestingly, cellular localisation of the *Hydra* Riox2 is also slightly different to the human protein. While human RIOX2 shows nucleoplasmic localisation with distinct accumulation in nucleoli (Fig. 2) [27, 28], the *Hydra* orthologue is a nuclear protein but seem to exclude nucleoli (Fig. 3). However, the conserved active site residues imply a fully functional enzyme. Further detailed molecular analyses would be necessary to unravel the enzymatic activity of *Hydra* Riox2. The original described activity of the human RIOX2 protein was demethylation of histone 3 lysine 9 tri-methylated (H3K9me3) residues [47], however, the evidence for this assignment is controversial [48]. Whether or not Riox2 might have dual functionality as a ribosomal oxygenase and/or as a histone demethylase needs to be separately investigated in each species which possesses an orthologous gene. Such studies should be further supported with structural approaches, such as those described for another *Hydra* 2OG oxygenase, Jmjd6 [49–51]. It is likely that throughout evolution Riox2 proteins have acquired different or additional substrate targets and hydroxylation activities.

## Conclusions

In conclusion, our phylogenetic and genomic analysis of Riox1 and Riox2 has revealed the maintenance of a unique protein architecture with conservation of active enzymatic sites throughout evolution in the whole animal kingdom. This strongly suggests that both orthologues have a consistent enzymatic function as Fe(II) and 2OG-dependent dioxygenases with likely ribosomal protein hydroxylation as their main function. However, at the genomic level both orthologs show diversifications in the evolution of their gene architectures and presence or absence of the *Riox2* gene in different taxa. Many higher vertebrates and certain fly species (e.g. *Drosophila*) possess an intronless *Riox1* orthologue, while *Riox2* is absent in most invertebrates. This suggests that Riox1 is the evolutionary older JmjC-domain-containing protein with ribosomal oxidase function. The more complex gene structure of *Riox1* with multiple exons and introns in lower marine metazoans suggests a different, perhaps, more complex regulation of protein expression in these organisms. If this hypothesis is true, that complex *Riox*1 gene structures and expression regulation correlates with adaptation to different environments remains to be determined by future research. Differences in expression of protein isoforms and/or subcellular localisations of Riox orthologs in different species, as shown here for *Hydra*, may be explained by different functional requirements and evolutionary lifestyle adaptation of different taxa.

## Methods

### Molecular cloning

Human full-length and truncated RIOX1 and RIOX2 sequences were sub-cloned into the mammalian expression plasmid pEGFP-N1 (Clontech). *Hydra Riox1* and *Riox2* were amplified from cDNA (*Hydra vulgaris*, total RNA extracted from whole animals, primer: hyNO66_NheF 5’-CAGGCTAGCATGAATAACAACAAAGTATCAGC-3′, hyNO66_XmaR 5’-GACCCGGGTGTATGGACCAATGGAACC-3′ for *Riox1*, and hyMina53_NheF CAGGCTAGCATGGTGAAACGCAAAGGTTC, hyMina53_XmaR GGCCCGGGTTTGATTTCAATCAAATCATCAC, for *Riox2*, respectively) and sub-cloned into the *Hydra* eGFP expression plasmid pHotG [31] by using the *Nhe*1 and *Xma*1 restriction sites.

### Cell culture and transfection

HeLa cells (ATCC; CCL-2) were cultured in Dulbecco’s modified Eagle’s medium (DMEM) supplemented with 10% fetal calf serum (FCS), penicillin (100 Uml^− 1^) and streptomycin (100 μgml^− 1^) at 37 °C, 5% CO_2_. Cells were transfected with expression constructs using Lipofectamine 2000 (Invitrogen) according to manufacturer’s instructions.

### Hydra culture

*Hydra vulgaris* strain Basel [52] were held in mass culture in hydra medium (0,1 mM KCL, 1 mM NaCl, 0,1 mM MgSO_4_, 1 mM Tris, 1 mM CaCl_2_), at a constant temperature of 18 °C and were fed regularly with freshly hatched *Artemia nauplii*.

### Transfection of Hydra cells

2,4 mg Gold particles (1,0 μm, BioRad) were coated with 10 μg plasmid DNA according to instructions of manufacturer. They were introduced into the Hydra cells with a Helios gene gun system (BioRad) as described [31].

### Fixation and mounting of *Hydra*

Animals were relaxed in 2% urethane in hydra medium and fixed with 4% paraformaldehyde (in PBS) at room temperature for one hour. After three washes with PBS, they were counterstained for DNA with DAPI (Sigma, 1 μg/ml) and mounted on slides with Vectashield mounting medium (Axxora).

### Confocal imaging of *Hydra*

Light optical sections were acquired with a Leica TCS SP5–2 confocal laser-scanning microscope. Fluorochromes were visualised with the 405 laser with an excitation wavelength of 405 nm and emission filters 413 to 443 nm for DAPI. The argon laser with excitation wavelength of 488 nm and emission filters 496 to 537 nm was used for GFP. Image resolution was 512 × 512 pixel. To obtain an improved signal-to-noise ratio, each section image was averaged from three successive scans.

### Immunostaining and microscopy of Hela cells

HeLa cells were grown to 50–70% confluence on 18 mm diameter coverslips. 24 h post-transfection cells were fixed with 4% paraformaldehyde (10 min). GFP-expressing cells were stained with 1 μg/ml DAPI and slides mounted in Vectashield. For antibody staining cells were permeabilized after fixation with 1% Triton X-100 in PBS and subsequently kept in blocking solution for an hour (10%FCS, 0.2% Tween-20 in PBS). Primary antibodies: α-Riox1 (Mina53, ab169154, Abcam), α-Riox1 (NO66, ab113975, Abcam) and α-UBF (sc-9131, Santa Cruz). Secondary antibodies: Alexa Fluor 488 chicken anti-mouse (A21200, Thermo Fisher), Alexa Fluor 594 donkey anti-rabbit (A21206, Thermo Fisher). Slides were imaged with a fluorescence microscope Carl Zeiss LSM 510 META.

### Ensemble database searches

Orthologes of *Riox1* and *Riox2* genes were identified using Ensembl (http://www.ensembl.org/info/about/species.html) and EnsemblMetazoa (http://metazoa.ensembl.org/species.html) portals by employing pblast searches. As described in the result section, the genome of each selected species was queried with the human or *Hydra vulgaris* RIOX1/Riox1 (NP_078920.2 and XP_002157896.3) and ROX2/Riox2 (NP_694822.2 and XP_002167270) protein reference sequences, respectively. For phylogenetic analyses of orthologues proteins only sequences were included in which both, the human and hydra pblast queries, matched to a single locus of the selected species and the identified protein displayed the characteristic domain architecture of Riox1 and Riox2 proteins as described below in the results section. Species were selected throughout the animal kingdom to represent main taxonomic classes, where available with at least two species per class depending on genome sequence coverage and quality of gene structure annotations. *Hydra vulgaris* protein sequences and gene annotations for *Riox1* and *Riox2* were obtained from https://metazome.jgi.doe.gov/pz/portal.html.

### Multiple-sequence alignments

FASTA-formatted amino-acid sequences were aligned using the MAFFT 7 tool (http://www.ebi.ac.uk/Tools/msa/mafft/) provided by the European Bioinformatics Institute (EBI) using the ClustalW algorithm [53]. The resulting multiple aa sequence alignment was used to generate phylogenetic trees.

### Construction of phylogenetic trees

To analyse the phylogenetic relationship of 41 Riox1 proteins from different species and to infer the evolutionary relationship of Ribosomal oxygenases, the JmjC domain sequences of Riox1 (No66) and Riox2 (Mina53) from 49 species and of ycfD (ecycfD), the ROX protein from *E. coli* [6], were subjected to a maximum likelihood analysis using the online phylogenetic tool W-IQ-TREE (Version 1.5.4 at http://iqtree.cibiv.univie.ac.at) [54]. In the IQ-Tree webserver the ‘Substitution model’ and the default ‘Auto’ settings were selected to determine the best-fit substitution model followed by tree construction. Within the ‘Branch Support Analysis’ the default settings of an ultrafast bootstrap analysis with 1000 replicates was used, with maximum number of iterations set at 1000 and a minimum correlation coefficient of 0.99 and for the ‘Single branch tests’ the SH-aLRT branch test with 1000 replicates was selected [55].

### Bioinformatic prediction of nuclear localisation sites

Nuclear localisation signal searches have been performed with NLS Mapper (http://nls-mapper.iab.keio.ac.jp/cgi-bin/NLS_Mapper_form.cgi) [34] and NLStradamus (http://www.moseslab.csb.utoronto.ca/NLStradamus/) [33].

## Additional files

Additional file 1: Protein sequence alignment (Clustal Omega) [35] of RIOX1 (*H.sapiens*) and Riox1 (*M.musculus*). The proposed iron-binding motif (H340, D342, H405) and the 2OG–interacting K355 for the human sequence [16] are indicated in green or blue respectively. (PDF 68 kb)

Additional file 2: Protein sequence alignment (Clustal Omega) [35] of RIOX1 (*H.sapiens*) and Riox1 (*G.gallus*). The proposed iron-binding motif (H340, D342, H405) and the 2OG–interacting K355 for the human sequence [16] are indicated in green or blue respectively. (PDF 106 kb)

Additional file 3: Protein sequence alignment (Clustal Omega) [35] of RIOX1 (*H.sapiens*) and Riox1 (*D.rerio*). The proposed iron-binding motif (H340, D342, H405) and the 2OG–interacting K355 for the human sequence [16] are indicated in green or blue respectively. (PDF 106 kb)

Additional file 4: Protein sequence alignment (Clustal Omega) [35] of RIOX1 (*H.sapiens*) and Riox1 (*D.melanogaster*). The proposed iron-binding motif (H340, D342, H405) and the 2OG–interacting K355 for the human sequence [16] are indicated in green or blue respectively. (PDF 103 kb)

Additional file 5: Protein sequence alignment (Clustal Omega) [35] of RIOX1 (*H.sapiens*) and Riox1 (*C.elegans*). The proposed iron-binding motif (H340, D342, H405) and the 2OG–interactingK355 for the human sequence [16] are indicated in green or blue respectively. (PDF 103 kb)

Additional file 6: Protein sequence alignment (Clustal Omega) [35] of RIOX2 (*H.sapiens*) and Riox2 (*M.musculus*). The proposed iron-binding motif (H179, D181, H240) and the 2OG–interacting K194 for the human sequence [16] are indicated in green or blue respectively. (PDF 68 kb)

Additional file 7: Protein sequence alignment (Clustal Omega) [35] of RIOX2 (*H.sapiens*) and Riox2 (*G.gallus*). The proposed iron-binding motif (H179, D181, H240) and the 2OG–interacting K194 for the human sequence [16] are indicated in green or blue respectively. (PDF 67 kb)

Additional file 8: Protein sequence alignment (Clustal Omega) [35] of RIOX2 (*H.sapiens*) and Riox2 (*D.rerio*). The proposed iron-binding motif (H179, D181, H240) and the 2OG–interacting K194 for the human sequence [16] are indicated in green or blue respectively. (PDF 68 kb)

Additional file 9: Nucleotide sequence (open reading frame) and corresponding aa sequence of Riox2 (*H.vulgaris*). Protein sequence alignment (Clustal Omega) [35] of RIOX2 (*H.sapiens*) and Riox2 (*H.vulgaris*). The proposed iron-binding motif (H179, D181, H240) and the 2OG–interacting K194 for the human sequence [16] are indicated in green or blue respectively. An additional stretch of charged aa in the *Hydra* sequence is highlighted in red. (PDF 71 kb)

Additional file 10: Nucleotide sequence (open reading frame) and corresponding aa sequence of Riox1 (*H.vulgaris*). Protein sequence alignment (Clustal Omega) [35] of RIOX1 (*H.sapiens*) and Riox1 (*H.vulgaris*). The proposed iron-binding motif (H340, D342, H405) and the 2OG–interacting K355 for the human sequence [16] are indicated in green or blue respectively. (PDF 71 kb)

Additional file 11: Clustal omega alignment of Riox1 protein sequences from *C.elegans*, *D.melanogaster*, *H.vulgaris*, zebrafish, chicken, mouse and human. The protein domains JmjC (red), dimerization (brown) and winged-helix (blue) are indicated based on the human sequence [16]. Lengths of the individual N-terminal extension domains are indicated (grey). The prospective iron-binding motif HxD…H (green) and the 2OG C5-carboxylate-binding residue (K, purple) are conserved in all species. Crystal structure analysis of human RIOX1 with substrate Rpl8 identified R297, Y328 and S421 residues of human RIOX1 involved in Rpl8 peptide binding [16] (red). (PDF 93 kb)

Additional file 12: Clustal omega alignment of Riox2 protein sequences from *H.vulgaris*, zebrafish, chicken, mouse and human. The protein domains JmjC (red), dimerization (brown) and winged-helix (blue) are indicated based on the human sequence [16]. The prospective iron-binding motif HxD…H (green) and the 2OG C5-carboxylate-binding residue (K, purple) are conserved in all species. Crystal structure analysis of human RIOX2 with substrate Rpl27a identified the aa N101, Q136, Q139, N165 and S257 of human RIOX2 involved in Rpl27a peptide binding [16] (red). (PDF 90 kb)

Additional file 13: Clustal omega alignment of Rpl8 (A) and Rpl27a (B) protein sequences from *C. intestinalis*, *H. vulgaris*, *C. elegans*, *A. melifera*, fruit fly, zebrafish, *X. laevis*, chicken, mouse and human. The protein sequence corresponds to the aa 208–224 of human RPL8 (A) and aa 30–51 of human RPL27A (B). Both aa-stretches harbour the H-residues, which get hydroxylated by human RIOX1 (H216 in RPL8) and RIOX2 (H39 in RPL27A). Sequences highlighted in red are species where no *Riox2* gene has been identified. (PDF 62 kb)

## Abbreviations

2OG: 2-oxoglutarate
D: Aspartate
E: Glutamate
G: Glycine
GFP: Green fluorescent protein
H: Histidine
JmjC: Jumonji C
K: Lysine
N: Asparagine
NLS: Nuclear localisation signal
NoLS: Nucleolar localisation signal
ORF: Open reading frame
Q: Glutamine
ROX: Ribosomal oxygenase
Th2: T helper 2
UTR: Untranslated region
WH: Winged-helix
Y: Tyrosine
