# Supplementary material for: Phylogenetic and genomic analyses of the ribosomal oxygenases Riox1 (No66) and Riox2 (Mina53) provide new insights into their evolution
Source: BMC Evol Biol. 2018 Jun 19;18:96. doi: 10.1186/s12862-018-1215-0 (PMC6006756; doi:10.1186/s12862-018-1215-0)
Supplement: Supplementary file 1 — Protein sequence alignment (Clustal Omega) [35] of RIOX1 (H.sapiens) and Riox1 (M.musculus). The proposed iron-binding motif (H340, D342, H405) and the 2OG–interacting K355 for the human sequence [16] are indicated in green or blue respectively. (PDF 68 kb) [file 12862_2018_1215_MOESM1_ESM.pdf]

Additional file 1: Figure S1

RIOX1 / NO66, *H.sapiens*: ENSG00000170468 (Ensembl)

Riox1 / No66, *M.musculus*: ENSMUSG00000046791 (Ensembl)

```
RIOX1 (H.sapiens)     MDGLQASA--GPLRRGRPKRR--RKPQPHSGSVLALPLRSRKIRKQLRSV-VSRMAALRT
Riox1 (M.musculus)    MDELPNGNGAALLKRGRGRRRRHPQSQPRGASVLALPLRPRKIRRHRKSAAASRVAALRA
                      ** *  .   . *:*** :**   : **:..******** ****:: :*. .**:****:

RIOX1 (H.sapiens)     QTLPSENSEESRVESTADDLGDALPGGAAVAAVPDAARREPYGHLGPAELLEASPAARSL
Riox1 (M.musculus)    RALRSEDSDSKVAV----------------ASVR-GK------RKRPAELLEASRSAEPR
                      ::* **:*:.. .                 *:*  .       :  ******** :*.

RIOX1 (H.sapiens)     QTPSARLVPASAPPARLVEVPAAPVRVVETSALLCTAQHLAAVQSSGAPATASGPQVDNT
Riox1 (M.musculus)    --------PVSARPRSA--SATLPSRVEGWAA-LS--RNLG----TAA-PPPPGSHADE-
                              *.** *       : * **   :* *.  :.*.    :.*     * :.*:

RIOX1 (H.sapiens)     GGEPAWDSPLRRVLAELNRIPSSRRRAARLFEWLIAPMPPDHFYRRLWEREAVLVRRQDH
Riox1 (M.musculus)    -PGRPRASPLQQVLTELNGIPSSRRRAARLFEWLLAPLPPDHFYRRLWEREAVLVRRQDR
                             ***::**:*** ****************:**:********************:

RIOX1 (H.sapiens)     TYYQGLFSTADLDSMLRNEEVQFGQHLDAARYINGRRETLNPPGRALPAAAWSLYQAGCS
Riox1 (M.musculus)    SYYEGLFSTADLDSMLRYEDVQFGQHLDAARYVDGRRETLNPPGRALPAAAWSLYRAGCS
                      :**:************* *:************::*********************:****
                                                                  H D            K
RIOX1 (H.sapiens)     LRLLCPQAFSTTVWQFLAVLQEQFGSMAGSNVYLTPPNSQGFAPHYDDIEAFVLQLEGRK
Riox1 (M.musculus)    LRLLCPQAFSPTVWQFLAVLQEQFGSMAGSNVYLTPPDSQGFAPHYDDIEAFVLQLEGRK
                      ********** **************************:**********************
                                                                       H
RIOX1 (H.sapiens)     LWRVYRPRVPTEELALTSSPNFSQDDLGEPVLQTVLEPGDLLYFPRGFIHQAECQDGVHS
Riox1 (M.musculus)    LWRVYRPRDPSEELALTSSPNFSQEDLGEPVLQTVLEPGDLLYFPRGFIHQAECQDGVHS
                      ******** *:*************:***********************************

RIOX1 (H.sapiens)     LHLTLSTYQRNTWGDFLEAILPLAVQAAMEENVEFRRGLPRDFMDYMGAQHSDSKDPRRT
Riox1 (M.musculus)    LHLTLSTYQRNTWGDFLEAVLPLAVQAAIEENVEFRRGLPRDFMDYMGAQHSDSKDPRRT
                      *******************:********:*******************************

RIOX1 (H.sapiens)     AFMEKVRVLVARLGHFAPVDAVADQRAKDFIHDSLPPVLTDRERALSVYGLPIRWEAGEP
Riox1 (M.musculus)    AFMEKVRVLVARLGHFAPVDAVADQRAKDFIHDSLPPVLTDRERALSVHGLPVRWEAGEP
                      ***********************************************:***:********

RIOX1 (H.sapiens)     VNVGAQLTTETEVHMLQDGIARLVGEGGHLFLYYTVENSRVYHLEEPKCLEIYPQQADAM
Riox1 (M.musculus)    VNVGAQLTTETQVHMLQDGVARLVGEGGRLFLYHTVENSRVYHLEEPKCLEIHPQQADAM
                      ***********:*******:********:****:******************:*******

RIOX1 (H.sapiens)     ELLLGSYPEFVRVGDLPCDSVEDQLSLATTLYDKGLLLTKMPLALN
Riox1 (M.musculus)    ELLLRSYPEFVRVGDLPCDSVEDQLSLATMLYDKGLLLTKTPLVPS
                      **** ************************ ********** **. .
```
