# Supplementary material for: Phylogenetic and genomic analyses of the ribosomal oxygenases Riox1 (No66) and Riox2 (Mina53) provide new insights into their evolution
Source: BMC Evol Biol. 2018 Jun 19;18:96. doi: 10.1186/s12862-018-1215-0 (PMC6006756; doi:10.1186/s12862-018-1215-0)
Supplement: Supplementary file 2 — Protein sequence alignment (Clustal Omega) [35] of RIOX1 (H.sapiens) and Riox1 (G.gallus). The proposed iron-binding motif (H340, D342, H405) and the 2OG–interacting K355 for the human sequence [16] are indicated in green or blue respectively. (PDF 106 kb) [file 12862_2018_1215_MOESM2_ESM.pdf]

Additional file 2: Figure S2

RIOX1 / NO66, *H.sapiens*: ENSG00000170468 (Ensembl)

Riox1 / No66, G.gallus: ENSGALG00000020454 (Ensembl)

```
RIOX1 (H.sapiens)   -----------------MDGLQASAGPLRRGRPKRRRKPQPHSGSVLALPLRSRKIRKQL
Riox1 (G.gallus)    MAACGAEERQRTGRLSALSVYRRAAGPGRLER-RRRGTPLPAGGKRTK---------ARL
                                     :.  : :*** *  * :** .* * .*.             :*

RIOX1 (H.sapiens)   RSVVSRMAALRTQTLPSENSEESRVESTADDLGDALPGGAAVAAVPDAAR-REPYGHLGP
Riox1 (G.gallus)    R-------------------------------RGAAGGGGPERAAPPQGAAVSDRVERAGS
                    *                               * *  **   ** *:.*   :   : *

RIOX1 (H.sapiens)   AEL----LEASPAARSLQTPSARLVPASAPPARLV-EVPAAPVRVVETSALLCTAQHLAA
Riox1 (G.gallus)    SEAKQGDLKTNPSGP----------PAKAPAGNLETKSPGGPLT---------TSVGPRA
                    :*     *::.*:.           **.** ..*  : *..*:          *:    *

RIOX1 (H.sapiens)   VQSSGAPAT-ASGPQVDNTGGEPAWDSPLRRVLAELNRIPSSRRRAARLFEWLIAPMPPD
Riox1 (G.gallus)    KAPAGSPEAKPPGDPATSPG-----GGGVPGLLRRLGRLEDSRRRAAELFRWLVAPAAPE
                       :*:* :   *  . . *      . :  :* .* *: .******.**.**:**  *:

RIOX1 (H.sapiens)   HFYRRLWEREAVLVRRQDHTYYQGLFSTADLDSMLRNEEVQFGQHLDAARYINGRRETLN
Riox1 (G.gallus)    EFARQHWERAPLLVQRGDPGYYAGLFSTADFDAILRSGDVHFGTHLDVTSYAEGVRETHN
                    .* *: ***  :**:* *  ** *******:*::**. :*:** ***.: * :* *** *

RIOX1 (H.sapiens)   PPGRALPAAAWSLYQAGCSLRLLCPQAFSTTVWQFLAVLQEQFGSMAGSNVYLTPPNSQG
Riox1 (G.gallus)    PVGRALPAVVWDFYQNGCSLRLLSPQAFSTTVWHFLSILQEHFGSMAGANTYLTPPGTQG
                    * ******..*.:** *******.*********:**::***:******:*.***** :**
                       H D            K
RIOX1 (H.sapiens)   FAPHYDDIEAFVLQLEGRKLWRVYRPRVPTEELALTSSPNFSQDDLGEPVLQTVLEPGDL
Riox1 (G.gallus)    FAPHYDDIEAFVLQLEGKKHWRVYGPRTSSEALPQFSSANLTQAELGEPLLEVVLEAGDL
                    *****************:* **** **. :* *   ** *::* :****:*:.*** ***
                            H
RIOX1 (H.sapiens)   LYFPRGFIHQAECQDGVHSLHLTLSTYQRNTWGDFLEAILPLAVQAAMEENVEFRRGLPR
Riox1 (G.gallus)    LYFPRGFIHQADCLPDAHSLHITVSSYQRNSWGDFLEKLLPAALQMALEEDLEYRQGLPM
                    ***********:*   .****:*:*:****:****** :** *:* *:**::*:*:***

RIOX1 (H.sapiens)   DFMDYMGAQHSDSKDPRRTAFMEKVRVLVARLGHFAPVDAVADQRAKDFIHDSLPPVLTD
Riox1 (G.gallus)    DCLGYMGVANSDAVDARRTAFVEKVQHLIKKLVDYAPIDAAMDQRAKSFLHDCLPPVLTQ
                    * : ***. .**: * *****:***: *: :* .:**:**. *****.*:**.******:

RIOX1 (H.sapiens)   RERALSVYGLPIRWEAGEPVNVGAQLTTETEVHMLQDGIARLVGEGGHLFLYYTVENSRV
Riox1 (G.gallus)    SEKQLSVYGFPARWQDGGPRNVDIQITKDTEVRLLHHGVVRLCNEEAGVMLYYTTENSRV
                     *: *****:* **: * * **  *:*.:***::*:.*:.**  * . ::****.*****

RIOX1 (H.sapiens)   YHLEEPKCLEIYPQQADAMELLLGSYPEFVRVGDLPCDSVEDQLSLATTLYDKGLLLTKM
Riox1 (G.gallus)aAAS YHKEEPKYLEIDPEYTDSIEFLLSSYPNHVSVDTLPCDALEDKISLATLLFEKGILTTKK
                    ** **** *** *: :*::*:**.***:.* *  ****::**::**** *::**:* **

RIOX1 (H.sapiens)   PLALN
Riox1 (G.gallus)    PLVQV
                    **.
```
