# Supplementary material for: Phylogenetic and genomic analyses of the ribosomal oxygenases Riox1 (No66) and Riox2 (Mina53) provide new insights into their evolution
Source: BMC Evol Biol. 2018 Jun 19;18:96. doi: 10.1186/s12862-018-1215-0 (PMC6006756; doi:10.1186/s12862-018-1215-0)
Supplement: Supplementary file 3 — Protein sequence alignment (Clustal Omega) [35] of RIOX1 (H.sapiens) and Riox1 (D.rerio). The proposed iron-binding motif (H340, D342, H405) and the 2OG–interacting K355 for the human sequence [16] are indicated in green or blue respectively. (PDF 106 kb) [file 12862_2018_1215_MOESM3_ESM.pdf]

Additional file 3: Figure S3

RIOX1 / NO66, *H.sapiens*: ENSG00000170468 (Ensembl)

Riox1 / No66, *D.rerio*: ENSDARG00000067838 (Ensembl)

```
Riox1 (D.rerio)        ------------------------------------------------------------
RIOX1 (H.sapiens)      MDGLQASAGPLRRGRPKRRRKPQPHSGSVLALPLRSRKIRKQLRSVVSRMAALRTQTLPS

Riox1 (D.rerio)        ----MERKHMSALSIYQSLSGGKTP-----------------------------------
RIOX1 (H.sapiens)      ENSEESRVESTADDLGDALPGGAAVAAVPDAARREPYGHLGPAELLEASPAARSLQTPSA
                            .* . :* .: ::* ** :

Riox1 (D.rerio)        -QAEDKAPPAKKVKRKENGVRPSKKATKKKGTKPLKSSVRSSSSEKEKHEGERDCREMNG
RIOX1 (H.sapiens)      RLVPASAPPARLVEVPAAPVRVVETSALLCTAQHL-AAVQSSG--APATASGPQVDNTGG
                         .  .****: *:     **  :.::    :: * ::*:**.       .  :  :  *

Riox1 (D.rerio)        KRFDNVALDILLTDLAKVNNSRDRANRLFQWLIHPVQDKSFFRDNWEKKPILIQRQNADY
RIOX1 (H.sapiens)      EPAWDSPLRRVLAELNRIPSSRRRAARLFEWLIAPMPPDHFYRRLWEREAVLVRRQDHTY
                       :   :  *  :*::* :: .** ** ***:*** *:  . *:*  **:: :*::**:  *

Riox1 (D.rerio)        YKGLFSTAEFDRILRNDDVQYGVNLDVTSYTNGKRETHNPPGRALPYTVWDFYESGCSIR
RIOX1 (H.sapiens)      YQGLFSTADLDSMLRNEEVQFGQHLDAARYINGRRETLNPPGRALPAAAWSLYQAGCSLR
                       *:******::* :***::**:* .**.: * **:*** ******** :.*.:*::***:*
                                                                 H D             K
Riox1 (D.rerio)        MLNPQAFSSTVWQVLSVLQEKFGSMAGANVYLTPPGTQGFAPHFDDIEAFVVQLEGRKHW
RIOX1 (H.sapiens)      LLCPQAFSTTVWQFLAVLQEQFGSMAGSNVYLTPPNSQGFAPHYDDIEAFVLQLEGRKLW
                       :* *****:****.*:****:******:******* :******:*******:****** *
                                                                      H
Riox1 (D.rerio)        RVYNPRCEDEVLSLVSSPNFSQDEIGEPVMDVVLEAGDLLYFPRGFVHQGDCLPDAHSLH
RIOX1 (H.sapiens)      RVYRPRVPTEELALTSSPNFSQDDLGEPVLQTVLEPGDLLYFPRGFIHQAECQDGVHSLH
                       ***.**   * *:*.********::****::.*** **********:**.:*   .****

Riox1 (D.rerio)        ITISSYQRNSWGDLMLKLMPAALEVAMEEDVEFRKGLPLDYLQYMGVQNSEKEDPRRDRF
RIOX1 (H.sapiens)      LTLSTYQRNTWGDFLEAILPLAVQAAMEENVEFRRGLPRDFMDYMGAQHSDSKDPRRTAF
                       :*:*:****:***::  ::* *::.****:****:*** *:::***.*.*:.:****  *

Riox1 (D.rerio)        MAHIQGLMKKLVSFAPVDAAVDQKAKDFLHDCLPPLLTAEEKAGSVYGAPARWGDSEALD
RIOX1 (H.sapiens)      MEKVRVLVARLGHFAPVDAVADQRAKDFIHDSLPPVLTDRERALSVYGLPIRWEAGEPVN
                       * ::: *: :*  ******..**:****:**.***:** .*:* **** * **  .* ::

Riox1 (D.rerio)        VAVELKSQTRIKLVRAGAARLCSDGDTVHLYYTTENSRVYHKEASKSFEMKTEHIDAMEF
RIOX1 (H.sapiens)      VGAQLTTETEVHMLQDGIARLVGEGGHLFLYYTVENSRVYHLEEPKCLEIYPQQADAMEL
                       *..:*.::*.::::: * *** .:*  :.****.******* *  *.:*:  :: ****:

Riox1 (D.rerio)Aa      LIHSYPKFVSVASLPCETAEAKMSLAELLFEKGLIFTAEPLTAQ
RIOX1 (H.sapiens)      LLGSYPEFVRVGDLPCDSVEDQLSLATTLYDKGLLLTKMPLALN
                       *: ***:** *..***::.*  ::***  *::***::*  **: :
```
