# Supplementary material for: Phylogenetic and genomic analyses of the ribosomal oxygenases Riox1 (No66) and Riox2 (Mina53) provide new insights into their evolution
Source: BMC Evol Biol. 2018 Jun 19;18:96. doi: 10.1186/s12862-018-1215-0 (PMC6006756; doi:10.1186/s12862-018-1215-0)
Supplement: Supplementary file 4 — Protein sequence alignment (Clustal Omega) [35] of RIOX1 (H.sapiens) and Riox1 (D.melanogaster). The proposed iron-binding motif (H340, D342, H405) and the 2OG–interacting K355 for the human sequence [16] are indicated in green or blue respectively. (PDF 103 kb) [file 12862_2018_1215_MOESM4_ESM.pdf]

Additional file 4: Figure S4

RIOX1 / NO66, *H.sapiens*: ENSG00000170468 (Ensembl)

Riox1 / No66, *D.melanogaster*: FBgn0266570 (Ensembl)

```
Riox1 (D.melanogaster)   MKKATTSAAAKSQGNSKMQKNANNGTAKDKKKPNLDKESNDSNSVSDMLAVT--KDQEVH
RIOX1 (H.sapiens)        MDGLQASAGP-----------LRRGRPKRRRKPQPHS-----GSVLALPLRSRKIRKQLR
                         *.   :**.             ..*  * ::**: ..      **  :   :    ::::

Riox1 (D.melanogaster)   AFFSKLFD--DDAGPSTSKKTQSGSAAAAKTADRKRRLQAEADANNNDTGKAGKLTKESE
RIOX1 (H.sapiens)        SVVSRMAALRTQTLPSEN--------------SEESRVESTA----DDLGDA--LPGGAA
                         :..*::     :: ** .              ..: *::: *    :* *.*  *   :

Riox1 (D.melanogaster)   ATQGARATKRKQARSLGL---ERTSPIQVNGAALACPLVRKSLPPGEANSCPQPPKKDPA
RIOX1 (H.sapiens)        VAAVPDAARREPYGHLGPAELLEASPAARSLQTPSARLVPASAPPARLVEVPAAPVRVVE
                         .:    *::*:    **     .:**   .  : :. **  * **..  . *  * :

Riox1 (D.melanogaster)   AVNSLV------KI--IKAEPTEEGNNN--------NDEK--ETETIETHKADSVEEGRR
RIOX1 (H.sapiens)        TSALLCTAQHLAAVQSSGAPATASGPQVDNTGGEPAWDSPLRRVLAELNRIPSSRRRAAR
                         :   *        :    *  * .* :          *.   .. :  .:  .* ... *

Riox1 (D.melanogaster)   VLQWILFPVQTKVFFKDFWEHTACLVQRSNPKYFQSMISFKMLDEILIRHHLDFTVNVDV
RIOX1 (H.sapiens)        LFEWLIAPMPPDHFYRRLWEREAVLVRRQDHTYYQGLFSTADLDSMLRNEEVQFGQHLDA
                         :::*:: *:  . *:: :**: * **:*.: .*:*.::*   **.:* ...::*  .:*.

Riox1 (D.melanogaster)   TTYKNGKRETLNPEGRALPPAVWGFYSDGCSIRLLNPSTYLIRLRQVCTVLQEFFHCKVG
RIOX1 (H.sapiens)        ARYINGRRETLNPPGRALPAAAWSLYQAGCSLRLLCPQAFSTTVWQFLAVLQEQFGSMAG
                         : * **:****** ***** *.*.:*. ***:*** *.::   : *. :**** * . .*
                                        H D                 K
Riox1 (D.melanogaster)   ANLYLTPPNSQGFAPHYDDIEAFVIQVEGRKRWLLYEPPKKADQLARISSGNYDQEQLGK
RIOX1 (H.sapiens)        SNVYLTPPNSQGFAPHYDDIEAFVLQLEGRKLWRVYRPRVPTEELALTSSPNFSQDDLGE
                         :*:*********************:*:**** * :*.*   ::**   ** *:.*::**:
                                             H
Riox1 (D.melanogaster)   PIIDEVLSAGDVLYFPRGAVHQAITEEQQHSLHITLSVYQQQAYANLLETLMPMVLKKAV
RIOX1 (H.sapiens)        PVLQTVLEPGDLLYFPRGFIHQAECQDGVHSLHLTLSTYQRNTWGDFLEAILPLAVQAAM
                         *::: **. **:****** :***  ::  ****:***.**::::.::**::*:.:: *:

Riox1 (D.melanogaster)   DRSVALRRGLPLHTFQVLGNAYKGNDCGSRKQLVENVQKLVTNYLMPSEDDIDEAVDQMA
RIOX1 (H.sapiens)        EENVEFRRGLPRDFMDYMGAQHSDSKDPRRTAFMEKVRVLVARL--GHFAPVDAVADQRA
                         :..* :***** . :: :*  :. ..   *. ::*:*: **:.        :* ..** *

Riox1 (D.melanogaster)   KKFQHEALPPIVLPSEEVRTVHGARSDADEQGNCVCDYKFNKKTSVRLLRANILRLVTES
RIOX1 (H.sapiens)        KDFIHDSLPPVLTDRERALSVYGLPIRWEAGEPVNVGAQLTTETEVHMLQDGIARLVGE-
                         *.* *::***::   *.. :*:*     :         ::..:*.*::*:  * *** *

Riox1 (D.melanogaster)A  DGSVRIYHHVDNGLDYCKYEPYFMEILPEEAKAVELLISAYPFYLTIDQLPLESSARKIE
RIOX1 (H.sapiens)        GGHLFLYYTVENSRVYHLEEPKCLEIYPQQADAMELLLGSYPEFVRVGDLPCDSVEDQLS
                          * : :*: *:*.  *   **   :** *::*.*:***:.:** :: : :** :*   ::.

Riox1 (D.melanogaster)   VATALWEHGLLMTEKPFK--
RIOX1 (H.sapiens)        LATTLYDKGLLLTKMPLALN
                         :**:*:::***:*: *:
```
