# Supplementary material for: Phylogenetic and genomic analyses of the ribosomal oxygenases Riox1 (No66) and Riox2 (Mina53) provide new insights into their evolution
Source: BMC Evol Biol. 2018 Jun 19;18:96. doi: 10.1186/s12862-018-1215-0 (PMC6006756; doi:10.1186/s12862-018-1215-0)
Supplement: Supplementary file 5 — Protein sequence alignment (Clustal Omega) [35] of RIOX1 (H.sapiens) and Riox1 (C.elegans). The proposed iron-binding motif (H340, D342, H405) and the 2OG–interactingK355 for the human sequence [16] are indicated in green or blue respectively. (PDF 103 kb) [file 12862_2018_1215_MOESM5_ESM.pdf]

Additional file 5: Figure S5

RIOX1 / NO66, *H.sapiens*: ENSG00000170468 (Ensembl)

Riox1 / No66, *C.elegans*: WBGene00020902 (Ensembl)

```
Riox1 (C. elegans)    MGKKKNSNKSAAAAPAVKHNDRWSSIELGEAKSAAVSHYKEPSKEPKFVHPAKLEKVKRI
RIOX1 (H.sapiens)     ------------------------------------------------------------


Riox1 (C. elegans)    HDGLNIDRVLSHGPVPKQNGGTKRKHVEVTTQKLENKKPKVEVKKEDEKSKNKKMKNQNK
RIOX1 (H.sapiens)     MDGLQA----SAGPLRR--GRPKRRRKPQPHS---G--SVLALP-----LRSRKIRKQLR
                       ***:     * **: :  *  **::     .        : :       :.:*:::* :

Riox1 (C. elegans)    HTALVQNETSTRSTYFVEEPDNENKVTLISNGREIAFKKTEVVESDDEQMIGLDSDEELE
RIOX1 (H.sapiens)     --SVVSRMAALRTQTLPSENSEESRVESTAD--------------------------DLG
                        ::*.. :: *:  : .* .:*.:*   ::                          :*

Riox1 (C. elegans)    DEDETDIDEDEMMIDPKDIERYINFESVEDEEDMEDEEIEDEEFED--EEFEDE-EEAD
RIOX1 (H.sapiens)     DA----LPGGAAVAAVPDAARREPYGHLGPAELLEASPA-ARSLQTPSARLVPASAPPAR
                      *     :     :    *  *   :  :   * :* .    ..::    .:       *

Riox1 (C. elegans)    EQE--EEEEDVSDEESVVSEMD--ADSDDEGF---IAGKDREAHVISKDKFTR--NAPAV
RIOX1 (H.sapiens)     LVEVPAAPVRVVETSALLCTAQHLAAVQSSGAPATASGPQVD-NTGGEPAWDSPLRRVLA
                        *       * : .:::.  :  *  :..*     :* : : .. .:  :    .   .

Riox1 (C. elegans)    DFDKFPFTDEDSVVTSSRAFGFMISPCDVQTFFDKFYQSNVLVVRRKQPTYFGNLFSTAR
RIOX1 (H.sapiens)     ELNRIP----SSRRRAARLFEWLIAPMPPDHFYRRLWEREAVLVRRQDHTYYQGLFSTAD
                      ::::*     .*   ::* * ::*:*   : *: :::: :.::***:: **:  *****

Riox1 (C. elegans)    LGELLEKNHLEYGRNINIAQYKNGVRTTLNGQGRAYPQIVKQHLHNMCSVQLVNPQTYDD
RIOX1 (H.sapiens)     LDSMLRNEEVQFGQHLDAARYINGRRETLNPPGRALPAAAWSLYQAGCSLRLLCPQAFST
                      * .:*.::.:::*:.:: *:* ** * ***  *** *  . .  :  **::*: **::.
                                                       H D            K
Riox1 (C. elegans)    RIWYLCEVIQEQFGCFVGANTYLTPAGSSGFAPHWDEIDAFLLQVEGRKYWRVWAPESAE
RIOX1 (H.sapiens)     TVWQFLAVLQEQFGSMAGSNVYLTPPNSQGFAPHYDDIEAFVLQLEGRKLWRVYRPRVPT
                       :* :  *:*****.:.*:*.****  *.*****:*:*:**:**:**** ***: *.
                                                            H
Riox1 (C. elegans)    EELPLESSDNFTEDDMKGREPVFEGWIEKGDMIYIPRGYIHQARTDSKVHSLHVTVSTGR
RIOX1 (H.sapiens)     EELALTSSPNFSQDDLG--EPVLQTVLEPGDLLYFPRGFIHQAECQDGVHSLHLTLSTYQ
                      *** * ** **::**:   ***::  :* **::*:***:****. :. *****:*:** :

Riox1 (C. elegans)    QWSFANLMEKVVPEAIGVLTDTRHKLRRGLPTGLFDMGGVIDLDYS--QEDHFVEKFKMV
RIOX1 (H.sapiens)     RNTWGDFLEAILPLAVQAAMEENVEFRRGLPRDFMDYMGAQHSDSKDPRRTAFMEKVRVL
                      : ::.:::* ::* *: .  : . ::*****  ::*  *. . * .  :.  *:**.:::

Riox1 (C. elegans)A   VDRHMSMLRNLVADQLLESSVDSLAKEFMKQALPPRLTEQEKKLSVLGSSTNLLGDDLVD
RIOX1 (H.sapiens)     VARLG-------HFAPVDAVADQRAKDFIHDSLPPVLTDRERALSVYGLPIRWEAGEPVN
                      * *             ::: .*. **:*::::*** **::*: *** *   .  . : *:

Riox1 (C. elegans)    FT----ARTKVRLIRRHTQRLLMESEDACFISHRINNSRLFEGRPEQIVEYPISGIDAYR
RIOX1 (H.sapiens)     VGAQLTTETEVHMLQDGIARLVG-EGGHLFLYYTVENSRVYHLEEPKCLEIYPQQADAME
                      .     :.*:*::::    **:  .    *: : ::***::. .  : :*   .  ** .

Riox1 (C. elegans)    VLSNSYPEWRTLYEIFSLRETKTKSRKENLAAIQLLFQIGVLLVKN-----
RIOX1 (H.sapiens)     LLLGSYPEFVRVGDLP------CDSVEDQLSLATTLYDKGLLLTKMPLALN
                      :*  ****:  : ::        .* ::*:     *:: *:**.*
```
