# Supplementary material for: Phylogenetic and genomic analyses of the ribosomal oxygenases Riox1 (No66) and Riox2 (Mina53) provide new insights into their evolution
Source: BMC Evol Biol. 2018 Jun 19;18:96. doi: 10.1186/s12862-018-1215-0 (PMC6006756; doi:10.1186/s12862-018-1215-0)
Supplement: Supplementary file 6 — Protein sequence alignment (Clustal Omega) [35] of RIOX2 (H.sapiens) and Riox2 (M.musculus). The proposed iron-binding motif (H179, D181, H240) and the 2OG–interacting K194 for the human sequence [16] are indicated in green or blue respectively. (PDF 68 kb) [file 12862_2018_1215_MOESM6_ESM.pdf]

Additional file 6: Figure S6

RIOX2 / MINA53, *H.sapiens*: ENSG00000170854 (Ensembl)

Riox2 / Mina53, M.musculus: ENSMUSG00000022724 (Ensembl)

```
Riox2 (M.musculus)   MPKKVQPTGDENEEASVPCKRVKEELPETLSVLNFDSPSSFFESLISPIKVETFFKEFWE
RIOX2 (H.sapiens)    MPKKAKPTGSGKEEGPAPCKQMKLEAAGGPSALNFDSPSSLFESLISPIKTETFFKEFWE
                     ****.:***. :**. .***::* *     *.********:*********.*********

Riox2 (M.musculus)   QKPLLIQRDDPVLAKYYQSLFSLSDLKRLCKKGVYYGRDVNVCRSISGKKKVLNKDGRAH
RIOX2 (H.sapiens)    QKPLLIQRDDPALATYYGSLFKLTDLKSLCSRGMYYGRDVNVCRCVNGKKKVLNKDGKAH
                     ***********.**.** ***.*:*** **.:*:**********.:.**********:**
                                                                               H
Riox2 (M.musculus)   FLQLRKDFDQKRATIQFHQPQRYKDELWRIQEKLECYFGSLVGSNVYMTPAGSQGLPPHY
RIOX2 (H.sapiens)    FLQLRKDFDQKRATIQFHQPQRFKDELWRIQEKLECYFGSLVGSNVYITPAGSQGLPPHY
                     **********************:************************:************
                     D            K                                               H
Riox2 (M.musculus)   DDVEVFILQLEGTKHWRLYSPTVPLAHEYSVESEDRIGTPTHDFLLKPGDLLYFPRGTIH
RIOX2 (H.sapiens)    DDVEVFILQLEGEKHWRLYHPTVPLAREYSVEAEERIGRPVHEFMLKPGDLLYFPRGTIH
                     ************ ****** ******:*****:*:*** *.*:*:***************

Riox2 (M.musculus)   QAETPSGLAYSIHLTISTYQNNSWGDCLLDSISGFVFDIAKEDVALRSGMPRRMLLNVET
RIOX2 (H.sapiens)    QADTPAGLAHSTHVTISTYQNNSWGDFLLDTISGLVFDTAKEDVELRTGIPRQLLLQVES
                     **:**:***:* *:************ ***:***:*** ***** **:*:**::**:**:

Riox2 (M.musculus)   PADVTRKLSGFLRTLADQLEGREELLSSDMKKDFVKHRLPPFFEGNGTETMDPGKQLPRL
RIOX2 (H.sapiens)    TTVATRRLSGFLRTLADRLEGTKELLSSDMKKDFIMHRLPPYSAGDGAELSTPGGKLPRL
                      : .**:**********:*** :***********: *****:  *:*:*   ** :****

Riox2 (M.musculus)   DNIIRLQFKDHIVLTVGPDKNPFDEAQQKVVYIYHSLKNVRQMHMIGEEEESEIFGLRFP
RIOX2 (H.sapiens)    DSVVRLQFKDHIVLTVLPDQDQSDEAQEKMVYIYHSLKNSRETHMMGNEEETEFHGLRFP
                     *.::************ **::  ****:*:********* *: **:*:***:*:.*****

Riox2 (M.musculus)   LSHVDALKQIWCGSPIRVKDLKLDTDEEKENLALSLWSESLIQVL
RIOX2 (H.sapiens)    LSHLDALKQIWNSPAISVKDLKLTTDEEKESLVLSLWTECLIQVV
                     ***:******* .  * ****** ******.*.****:*.****:
```
