# Supplementary material for: Phylogenetic and genomic analyses of the ribosomal oxygenases Riox1 (No66) and Riox2 (Mina53) provide new insights into their evolution
Source: BMC Evol Biol. 2018 Jun 19;18:96. doi: 10.1186/s12862-018-1215-0 (PMC6006756; doi:10.1186/s12862-018-1215-0)
Supplement: Supplementary file 7 — Protein sequence alignment (Clustal Omega) [35] of RIOX2 (H.sapiens) and Riox2 (G.gallus). The proposed iron-binding motif (H179, D181, H240) and the 2OG–interacting K194 for the human sequence [16] are indicated in green or blue respectively. (PDF 67 kb) [file 12862_2018_1215_MOESM7_ESM.pdf]

Additional file 7: Figure S7

RIOX2 / MINA53, *H.sapiens*: ENSG00000170854 (Ensembl)

Riox2 / Mina53, *G.gallus*: ENSGALG00000039302 (Ensembl)

```
RIOX2 (H.sapiens)   MPKKAKPTGSGKEEGPAPCKQMKLEAAGGPSALNFDSPSSLFESLISPIKTETFFKEFWE
Riox2 (G.gallus)    MPKKRGKHAEMGMEMQVQSKRAKVETDCSPSVMDFESPESLFASLISPIKPEVFFKEYWE
                    ****    ..   *  . .*: *:*:  .**.::*:**.*** ******* *.****:**

RIOX2 (H.sapiens)   QKPLLIQRDDPALATYYGSLFKLTDLKSLCSRGMYYGRDVNVCRCVNGKKKVLNKDGKAH
Riox2 (G.gallus)    EKPLLVQRNNPLLAAYYQSLFQLSDLKELCSQGLYYGRDINICRCVNGKKKVLNKEGKVN
                    :****:**::* **:** ***:*:***.***:*:*****:*:*************:**..

                                                                               H
RIOX2 (H.sapiens)   FLQLRKDFDQKRATIQFHQPQRFKDELWRIQEKLECYFGSLVGSNVYITPAGSQGLPPHY
Riox2 (G.gallus)    YAQLKKDFDQKKATIQFHQPQRFKEELWKIQEKLECYFGSLVGSNVYITPQGSQGLPPHY
                    : **:******:************:***:********************* *********

                    D            K                                             H
RIOX2 (H.sapiens)   DDVEVFILQLEGEKHWRLYHPTVPLAREYSVEAEERIGRPVHEFMLKPGDLLYFPRGTIH
Riox2 (G.gallus)    DDVEVFILQLEGEKHWRLYKPTVHLAREYNVESEDRIGNPTHEFVLKPGDLLYFPRGTIH
                    *******************:*** *****.**:*:***.*.***:***************

RIOX2 (H.sapiens)   QADTPAGLAHSTHVTISTYQNNSWGDFLLDTISGLVFDTAKEDVELRTGIPRQLLLQVES
Riox2 (G.gallus)    QADTPPGIPYSTHVTISTYQNNSWGDFLLDAIPGLVFSTAKDDVALRTSIPRKLLMQVDI
                    ***** *: :********************:* ****.***:** ***.***:**:**:

RIOX2 (H.sapiens)   TTVATRRLSGFLRTLADRLEGTKELLSSDMKKDFIMHRLPPYSAGDGAELSTPGGKLPRL
Riox2 (G.gallus)    AD-STKKLSSILRMLADRLENTGELRSSDMRKDFIMNRLPPCLGCDS-DSLTPGGKVPKL
                    :  :*::**.:** ****** * ** ****:*****.****  . *. :  *****:*:*

RIOX2 (H.sapiens)   DSVVRLQFKDHIVLTVLPDQDQSDEAQEKMVYIYHSLKNSRETHMMGNEEE---------
Riox2 (G.gallus)    DSKIRLQFRDHAVITVEPDQENSDEIRKEMVYVYHSLKNRRETHMMGTEDDDTGSEEGAA
                    ** :****:** *:** ***::*** :::***:****** *******.*::

RIOX2 (H.sapiens)   -TEFHGLRFPLSHLDALKQIWNSPAISVKDLKLTTDEEKESLVLSLWTECLIQVV
Riox2 (G.gallus)    QQTPHGLRFPLSYLDALKQIWSGSTVSVKELKLTSAEEKENLALALWTECLIEVF
                        ********:********.. ::***:****: ****.*.*:*******:*.
```
