# Supplementary material for: Phylogenetic and genomic analyses of the ribosomal oxygenases Riox1 (No66) and Riox2 (Mina53) provide new insights into their evolution
Source: BMC Evol Biol. 2018 Jun 19;18:96. doi: 10.1186/s12862-018-1215-0 (PMC6006756; doi:10.1186/s12862-018-1215-0)
Supplement: Supplementary file 8 — Protein sequence alignment (Clustal Omega) [35] of RIOX2 (H.sapiens) and Riox2 (D.rerio). The proposed iron-binding motif (H179, D181, H240) and the 2OG–interacting K194 for the human sequence [16] are indicated in green or blue respectively. (PDF 68 kb) [file 12862_2018_1215_MOESM8_ESM.pdf]

Additional file 8: Figure S8

RIOX2 / MINA53, *H.sapiens*: ENSG00000170854 (Ensembl)

Riox2 / Mina53, *D.rerio*: ENSDARG00000036359 (Ensembl)

```
Riox2 (D.rerio)      MPKISRRAARRSGQEERLQRVSSASSPATQRSGSPQEIEQEERLQRVSTLSSSGSPQEFF
RIOX2 (H.sapiens)    MPKKAKPT--GSGKEEG---------P------APCKQMKLE-AAGGPSALNFDSPSSLF
                     *** :: :   **:**          *      :* :  : *      :  .  **..:*

Riox2 (D.rerio)      QSLIRPLDLQEFFQRFWERQPLVLHRSDAALAGYYGSLFPLSGLRRLCARGLQYGTDINT
RIOX2 (H.sapiens)    ESLISPIKTETFFKEFWEQKPLLIQRDDPALATYYGSLFKLTDLKSLCSRGMYYGRDVNV
                     :*** *:. : **:.***::**:::*.* *** ****** *: *: **:**: ** *:*.

Riox2 (D.rerio)      CRCVRGQKRLLNRAGAVDFCLLERDFLEKKATIQFHQPQRFQDELWRIQERLECFFGCLV
RIOX2 (H.sapiens)    CRCVNGKKKVLNKDGKAHFLQLRKDFDQKRATIQFHQPQRFKDELWRIQEKLECYFGSLV
                     ****.*:*::**: * ..*  *.:** :*:***********:********:***:**.**

Riox2 (D.rerio)      GSNVYITPAGAQGLPPHYDDVEVLILQLEGQKHWRLYEPTVPLAREYSLEPEGRIGAPTH
RIOX2 (H.sapiens)    GSNVYITPAGSQGLPPHYDDVEVFILQLEGEKHWRLYHPTVPLAREYSVEAEERIGRPVH
                     **********:************:******:******.**********:* * *** *.*

Riox2 (D.rerio)      DFILQAGDLLYFPRGTIHQADTPAGAGHSTHLTLSTYQNMCVCAVHNVTHTHTHTLQNVL
RIOX2 (H.sapiens)    EFMLKPGDLLYFPRGTIHQADTPAGLAHSTHVTISTYQN---------------------
                     :*:*: ******************* .****:*:*****

Riox2 (D.rerio)      VLHSFIVCVRACRSWGDLLLDLMPGCVFDRMKTDCELRTGLPRGLLTTPSISPAVSHQLS
RIOX2 (H.sapiens)    ------------NSWGDFLLDTISGLVFDTAKEDVELRTGIPRQLLLQVESTTVATRRLS
                                 .****:*** : * ***  * * *****:** **   . : ..:::**

Riox2 (D.rerio)      VFLQRLADVVDHQGQTLRSSSMRRDFISHRLPPFVQD--PQLLQPVGGAPALQDTVSLRF
RIOX2 (H.sapiens)    GFLRTLADRLEGT-KELLSSDMKKDFIMHRLPPYSAGDGAELSTPGGKLPRLDSVVRLQF
                      **: *** ::   : * **.*::*** *****:      :*  * *  * *:..* *:*

Riox2 (D.rerio)      KDHLLLTVEPSPDHTDEATELLVYVLHSLRNRRDTHMMMGASDEDEDDEESQVGGLRFPL
RIOX2 (H.sapiens)    KDHIVLTVLPDQDQSDEAQEKMVYIYHSLKNSRETHMMGN-------EEETEFHGLRFPL
                     ***::*** *. *::*** * :**: ***:* *:****         :**::. ******

Riox2 (D.rerio)      SHLEALQQLLVSDRVPVEDLQLQQ-EDKLNLLLALWSEGLLRVTGALENHH
RIOX2 (H.sapiens)    SHLDALKQIWNSPAISVKDLKLTTDEEKESLVLSLWTECLIQVV-------
                     ***:**:*:  *  : *:**:*   *:* .*:*:**:* *::*.
```
