# Supplementary material for: Phylogenetic and genomic analyses of the ribosomal oxygenases Riox1 (No66) and Riox2 (Mina53) provide new insights into their evolution
Source: BMC Evol Biol. 2018 Jun 19;18:96. doi: 10.1186/s12862-018-1215-0 (PMC6006756; doi:10.1186/s12862-018-1215-0)
Supplement: Supplementary file 10 — Nucleotide sequence (open reading frame) and corresponding aa sequence of Riox1 (H.vulgaris). Protein sequence alignment (Clustal Omega) [35] of RIOX1 (H.sapiens) and Riox1 (H.vulgaris). The proposed iron-binding motif (H340, D342, H405) and the 2OG–interacting K355 for the human sequence [16] are indicated in green or blue respectively. (PDF 71 kb) [file 12862_2018_1215_MOESM10_ESM.pdf]

Additional file 10: Figure S10

Riox1 / No66 - *Hydra vulgaris*

Nucleotide sequence

ATGAATAACAACAAAGTATCAGCATTTAGTGTTTTTAAAGCTAAAGAAAAAATTGGTTTTGATATTGGTCTTGATTCACCTTCATC
AAAAAGTTTAAATACTCCAAGTAAAAATGGTGACATGAAATCTTCCAATGTTAAAAAAAAATTAAAACGCAATAAAAATGAGCCAC
TAAATTCATTACGTAATTCAATTCGTAAAAAAGTCAAAAAAGACGCACATCATGAGATATCATTAAAACCTGCAGAAAATAAGAGG
AAACCCTCTGTACTTCAAGTGTTTGAACGCAATTGGGCAGAAAATATTGCTGACTTAAACAGCTCCTCGTTAGAAAGGGAATTTTT
ACCTCAAGTTTTGGAACAAAAGAAGGTCCAAAATGACCTGCATAAAATAGGTTATCAAGAAAAATCTAATTCCGTTGTAGATTTAA
CAGCAAAAAAATCTGAAAAGAAAAAATCTATAAGATTAAAAAACCAAAGTTCAACAGCATCATTTGCTGCAATAAATCAAGAAAAA
AATGATATTACCAATGAACATTCAAAAAATAAAATTAAAAACTTAAGTTCTCTTTCTGGTAAAGAGTTGTTTAACTGGGTACTAGC
ACCAATAAGTTCTGAGAGATTTTTCAGTGAAGCATGGCAAAAAAAACCATTATTCATTAAAAGAAGGCAACCTCTTTACAACACCA
ATTGGTTCAGTACAAAAAAACTCGACAAAATTCTCAGAGAGAAAAATGTTCAATATACAAAAAATTTAGACATTGCAGTTTATAGA
AATGGACAACGTGAAACATTAAACCCTGAAGGGAGAGCTTTTCCATCTGTTGTCTGGAAGTTTTATGAAGATGGTTGTTCTATTCG
TCTTTTAAATCCACAAATATTTGCTAAAAGTGTCCATCAACTAACATCACGCCTTCAGGAGTATTTTGGTTGTTTAGTTGGATCTA
ATGTGTATTTGACACCACCTGGTAGTCAAGGGTTTGCACCGCACTATGATGATATTGAAGCTTTTGTTATTCAACTTGAAGGAAAA
AAACACTGGAAGCTTTATCCACCAAGAAACACAAATGAAGTTTTAGCTCGTTATTCTAGTGAAAACATGCAAGAAGAAAATTTAGG
TGAACCAATACTAAATAAAGTTCTAGAAGCTGGAGATACTCTTTATTTTCCAAGAGGTGTTATTCATCAAGCTTCCACACTTGAAG
ATTCCCATTCATTACATATTACTATATCTCTATATCAAAAAAGCTCTTGGGGTGATTATCTTGAGAAGCTTATCCCATTAGCATTA
CAAAAAGCAATTAGTGAAAACGTAATGTTCAGAGAAGGACTTCCTATTGATTTTTCTTCATTTGTTGGCGTTTCAAATTCAGAAAA
GAAATGTCCTGAGCGCGATACTTTTGTAAAAACAGTTAAAAAACTTATGGAGAAGCTTATTGATTATGTTGAAATTGATGAAGCAG
GCGATGAGCTCGTGTTGGATCACATGGAAGAATTTCAGCCACCGTACTATTCTTCTGATGATGTTGAATGCTCAGTGTTTAGTAAA
GATGGATTTTGGGATGGTGAAGTGCGACGTCATCTTAAAATAGAATTGAGTACTGAGTTAAGATTAGTTCGTCCAGGAATAGTTCG
GGTTATTCCAGCAGATACTGAGTTGCAAGTTTATTATTCTGTCGAAAACAGTCGGCAATATAAAGAAGTTCCTCTCAGAGTGCTTC
GTTTTTCACTAGAGGATGCCGACCTTTTGGAATATTTTTTGTTCAGTTATCCAGCGTACGTTGTTGTTGAAAACGCTCCTGGTGAT
GATATCCATAAGGTTGATGTCGCTAATCGTCTATACAACTTTGGTATTTTACGATCTAAGGTTCCATTGGTCCATAC

Amino acid sequence

MNNNKVSAFSVFKAKEKIGFDIGLDSPSSKSLNTPSKNGDMKSSNVKKKLKRNKNEPLNSLRNSIRKKVKKDAHHEISLKPAENKR
KPSVLQVFERNWAENIADLNSSSLEREFLPQVLEQKKVQNDLHKIGYQEKSNSVVDLTAKKSEKKKSIRLKNQSSTASSAAINQEK
NDITNEHSKNKIKNLSSLSGKELFNWVLAPISSERFFSEAWQKKPLFIKRRQPLYNTNWFSTKKLDKILREKNVQYTKNLDIAVYR
NGQRETLNHEGRAFPSVVWKFYEDGCSIRLLNPQIFAKSVHQLTSRLQEYFGCLVGSNVYLTPPGSQGFAPHYDDIEAFVIQLEGK
KHWKLYPPRNTNEVLARYSSENMQEENLGEPILNKVLEAGDTLYFPRGVIHQASTLEDSHSLHITISLYQKSSWGDYLEKLIPLAL
QKAISENVMFREGLPIDFSSFVGVSNSEKKCPERDTFVKTVKKLMEKLIDYVEIDEAGDELVLDHMEEFQPPYYSSDDVECSVFSK
DGFWDGEVRRHHKIELSTELRLVRPGIVRVIPADTELQVYYSVENSRQYKEVPLRVLRFSLEDADLLEYFLFSYPAYVVVENAPGD
DIHKVDVANRLYNFGILRSKVPLVHT

Alignment RIOX1 (*H. vulgaris* / *H. sapiens)*

```
Riox1 (H.vulgaris)     MNNNKVSAFSVFKAKEKIGFDIGLDSPSSKSLNTPSKNGDMKSSNVKKKLKRNKNEPLNS
RIOX1 (H.sapiens)      MDGLQASAGPL-----------RRGRPKRRRKPQPH-SGSVLALPLRS----------RK
                       *:. :.**  :            . *. :    *  .*.: :  ::.          ..

Riox1 (H.vulgaris)     LRNSIRKKVKKDAHHEISLKPAENKRKPSVL-----------------QVFERNWAENIA
RIOX1 (H.sapiens)      IRKQLRSVVSRMAALRTQTLPSENSEESRVESTADDLGDALPGGAAVAAVPDAARREPYG
                       :*:.:*. *.: *  . .  *:**..:  *                   * :    *  .

Riox1 (H.vulgaris)     DLNSS------SLER-------EFLPQVLEQKKVQNDLHKIGYQEKSNSVVDLTAKKSEK
RIOX1 (H.sapiens)      HLGPAELLEASPAARSLQTPSARLVPASAPPAR----LVE--VPAAPVRVVETSALLCTA
                       .*. :         *       .::*      :    * :         **: :*  .

Riox1 (H.vulgaris)     K--KSIRLKNQSSTASSAAINQEKNDIT-NEHSKNKI-----KNLSSLSGKELFNWVLAP
RIOX1 (H.sapiens)      QHLAAVQSSGAPATASGPQVDNTGGEPAWDSPLRRVLAELNRIPSSRRRAARLFEWLIAP
                       :   ::: ..  :***.  :::  .: : :.  :. :        *   . .**:*::**

Riox1 (H.vulgaris)     ISSERFFSEAWQKKPLFIKRRQPLYNTNWFSTKKLDKILREKNVQYTKNLDIAVYRNGQR
RIOX1 (H.sapiens)      MPPDHFYRRLWEREAVLVRRQDHTYYQGLFSTADLDSMLRNEEVQFGQHLDAARYINGRR
                       :  ::*: . *::: ::::*::  *  . *** .**.:**:::**: ::** * * **:*

Riox1 (H.vulgaris)     ETLNHEGRAFPSVVWKFYEDGCSIRLLNPQIFAKSVHQLTSRLQEYFGCLVGSNVYLTPP
RIOX1 (H.sapiens)      ETLNPPGRALPAAAWSLYQAGCSLRLLCPQAFSTTVWQFLAVLQEQFGSMAGSNVYLTPP
                       ****  ***:*:..*.:*: ***:*** ** *:.:* *: : *** **.:.*********
                              H D              K
Riox1 (H.vulgaris)     GSQGFAPHYDDIEAFVIQLEGKKHWKLYPPRNTNEVLARYSSENMQEENLGEPILNKVLE
RIOX1 (H.sapiens)      NSQGFAPHYDDIEAFVLQLEGRKLWRVYRPRVPTEELALTSSPNFSQDDLGEPVLQTVLE
                       .****************:****:* *::* **  .* **  ** *:.:::****:*:.***
                                 H
Riox1 (H.vulgaris)     AGDTLYFPRGVIHQASTLEDSHSLHITISLYQKSSWGDYLEKLIPLALQKAISENVMFRE
RIOX1 (H.sapiens)      PGDLLYFPRGFIHQAECQDGVHSLHLTLSTYQRNTWGDFLEAILPLAVQAAMEENVEFRR
                        ** ******.****.  :. ****:*:* **:.:***:** ::***:* *:.*** **.

Riox1 (H.vulgaris)     GLPIDFSSFVGVSNSEKKCPERDTFVKTVKKLMEKLIDYVEIDEAGDELVLDHMEEFQPP
RIOX1 (H.sapiens)      GLPRDFMDYMGAQHSDSKDPRRTAFMEKVRVLVARLGHFAPVDAVADQRAKDFIHDSLPP
                       *** ** .::*..:*:.* *.* :*::.*: *: :* .:. :* ..*: . *.:.:  **

Riox1 (H.vulgaris)     YYSSDDVECSVFSKDGFWDGEVRR--HHKIELSTELRLVRPGIVRVIPADTELQVYYSVE
RIOX1 (H.sapiens)      VLTDRERALSVYGLPIRWEAGEPVNVGAQLTTETEVHMLQDGIARLVGEGGHLFLYYTVE
                         :. :   **:.    *:.        ::  .**:::: **.*::  . .* :**:**

Riox1 (H.vulgaris)     NSRQYKEVPLRVLRFSLEDADLLEYFLFSYPAYVVVENAPGDDIH-KVDVANRLYNFGIL
RIOX1 (H.sapiens)      NSRVYHLEEPKCLEIYPQQADAMELLLGSYPEFVRVGDLPCDSVEDQLSLATTLYDKGLL
                       *** *:    : *.:  ::** :* :* *** :* * : * *.:. ::.:*. **: *:*

Riox1 (H.vulgaris)     RSKVPLVHT
RIOX1 (H.sapiens)      LTKMPLALN
                        :*:**. .
```
