# Supplementary material for: Phylogenetic and genomic analyses of the ribosomal oxygenases Riox1 (No66) and Riox2 (Mina53) provide new insights into their evolution
Source: BMC Evol Biol. 2018 Jun 19;18:96. doi: 10.1186/s12862-018-1215-0 (PMC6006756; doi:10.1186/s12862-018-1215-0)
Supplement: Supplementary file 11 — Clustal omega alignment of Riox1 protein sequences from C.elegans, D.melanogaster, H.vulgaris, zebrafish, chicken, mouse and human. The protein domains JmjC (red), dimerization (brown) and winged-helix (blue) are indicated based on the human sequence [16]. Lengths of the individual N-terminal extension domains are indicated (grey). The prospective iron-binding motif HxD…H (green) and the 2OG C5-carboxylate-binding residue (K, purple) are conserved in all species. Crystal structure analysis of human RIOX1 with substrate Rpl8 identified R297, Y328 and S421 residues of human RIOX1 involved in Rpl8 peptide binding [16] (red). (PDF 93 kb) [file 12862_2018_1215_MOESM11_ESM.pdf]

Additional file 11: Figure S11

```
                  N-terminal extension                          JmjC domain
C. elegans        286 aa                                       —AVDFDKFPFTDEDSVVTS
D. melanogaster   196 aa                                       —-KETETIETHKADSVEEG
H. vulgaris       179 aa                                       —SKNKI-----KNLSSLSG
H. sapiens        184 aa                                       —LRRVLAELNRIPSSRRRA
M. musculus       146 aa                                       —LQQVLTELNGIPSSRRRA
G. gallus         144 aa                                       —VPGLLRRLGRLEDSRRRA
D. rerio          87 aa                                        —LDILLTDLAKVNNSRDRA
                                                                            *    .

C. elegans        SRAFGFMISPCDVQTFFDKFYQSNVLVVRRKQPTYFGNLFSTARLGELLEKNHLEYGRNI
D. melanogaster   RRVLQWILFPVQTKVFFKDFWEHTACLVQRSNPKYFQSMISFKMLDEILIRHHLDFTVNV
H. vulgaris       KELFNWVLAPISSERFFSEAWQKKPLFIKRRQPLYNTNWFSTKKLDKILREKNVQYTKNL
H. sapiens        ARLFEWLIAPMPPDHFYRRLWEREAVLVRRQDHTYYQGLFSTADLDSMLRNEEVQFGQHL
M. musculus       ARLFEWLLAPLPPDHFYRRLWEREAVLVRRQDRSYYEGLFSTADLDSMLRYEDVQFGQHL
G. gallus         AELFRWLVAPAAPEEFARQHWERAPLLVQRGDPGYYAGLFSTADFDAILRSGDVHFGTHL
D. rerio          NRLFQWLIHPVQDKSFFRDNWEKKPILIQRQNADYYKGLFSTAEFDRILRNDDVQYGVNL
                   . : ::: *   . *    ::    .::* :  *    :*   :  :*   .:.:  .:

                                                      R/Q
C. elegans        NIAQYKNGVRTTLNGQGRAYPQIVKQHLHNMCSVQLVNPQTYDDRIWYLCEVIQEQFGCF
D. melanogaster   DVTTYKNGKRETLNPEGRALPPAVWGFYSDGCSIRLLNPSTYLIRLRQVCTVLQEFFHCK
H. vulgaris       DIAVYRNGQRETLNHEGRAFPSVVWKFYEDGCSIRLLNPQIFAKSVHQLTSRLQEYFGCL
H. sapiens        DAARYINGRRETLNPPGRALPAAAWSLYQAGCSLRLLCPQAFSTTVWQFLAVLQEQFGSM
M. musculus       DAARYVDGRRETLNPPGRALPAAAWSLYRAGCSLRLLCPQAFSPTVWQFLAVLQEQFGSM
G. gallus         DVTSYAEGVRETHNPVGRALPAVVWDFYQNGCSLRLLSPQAFSTTVWHFLSILQEHFGSM
D. rerio          DVTSYTNGKRETHNPPGRALPYTVWDFYESGCSIRMLNPQAFSSTVWQVLSVLQEKFGSM
                  : : * :* * * *  *** *  .         **:::: *. :    :  .   :** * .

                       Y            H D               K
C. elegans        VGANTYLTPAGSSGFAPHWDEIDAFLLQVEGRKYWRVWAPESAEEELPLESSDNFTEDDM
D. melanogaster   VGANLYLTPPNSQGFAPHYDDIEAFVIQVEGRKRWLLYEPPKKADQLARISSGNYDQEQL
H. vulgaris       VGSNVYLTPPGSQGFAPHYDDIEAFVIQLEGKKHWKLYPPRNTNEVLARYSSENMQEENL
H. sapiens        AGSNVYLTPPNSQGFAPHYDDIEAFVLQLEGRKLWRVYRPRVPTEELALTSSPNFSQDDL
M. musculus       AGSNVYLTPPDSQGFAPHYDDIEAFVLQLEGRKLWRVYRPRDPSEELALTSSPNFSQEDL
G. gallus         AGANTYLTPPGTQGFAPHYDDIEAFVLQLEGKKHWRVYGPRTSSEALPQFSSANLTQAEL
D. rerio          AGANVYLTPPGTQGFAPHFDDIEAFVVQLEGRKHWRVYNPRCEDEVLSLVSSPNFSQDEI
                  .*:* ****   :.*****:*:*:**::*:**:* * :: *     : *   ** *  : ::

                                        H              S     dimerization domain
C. elegans        KGREPVFEGWIEKGDMIYIPRGYIHQARTDSKVHSLHVTVSTGRQWSFANLMEKVVPEAI
D. melanogaster   --GKPIIDEVLSAGDVLYFPRGAVHQAITEEQQHSLHITLSVYQQQAYANLLETLMPMVL
H. vulgaris       --GEPILNKVLEAGDTLYFPRGVIHQASTLEDSHSLHITISLYQKSSWGDYLEKLIPLAL
H. sapiens        --GEPVLQTVLEPGDLLYFPRGFIHQAECQDGVHSLHLTLSTYQRNTWGDFLEAILPLAV
M. musculus       --GEPVLQTVLEPGDLLYFPRGFIHQAECQDGVHSLHLTLSTYQRNTWGDFLEAVLPLAV
G. gallus         --GEPLLEVVLEAGDLLYFPRGFIHQADCLPDAHSLHITVSSYQRNSWGDFLEKLLPAAL
D. rerio          --GEPVMDVVLEAGDLLYFPRGFVHQGDCLPDAHSLHITISSYQRNSWGDLMLKLMPAAL
                     :*:::  :. ** :*:***  :**.      ****:*:*  :: ::.: :  ::* .:

C. elegans        GVLTDTRHKLRRGLPTGLFDMGGVIDLD--YSQEDHFVEKFKMVVDRHMSMLRNLVADQL
D. melanogaster   KKAVDRSVALRRGLPLHTFQVLGNAYKGNDCGSRKQLVENVQKLVTNYLM-----PSEDD
H. vulgaris       QKAISENVMFREGLPIDFSSFVGVSNSEKKCPERDTFVKTVKKLMEKLI-------DYVE
H. sapiens        QAAMEENVEFRRGLPRDFMDYMGAQHSDSKDPRRTAFMEKVRVLVARLG-------HFAP
M. musculus       QAAIEENVEFRRGLPRDFMDYMGAQHSDSKDPRRTAFMEKVRVLVARLG-------HFAP
G. gallus         QMALEEDLEYRQGLPMDCLGYMGVANSDAVDARRTAFVEKVQHLIKKLV-------DYAP
D. rerio          EVAMEEDVEFRKGLPLDYLQYMGVQNSEKEDPRRDRFMAHIQGLMKKLV-------SFAP
                      .     *.***        *          .  ::  .: :: .

                                 winged helix domain
C. elegans        LESSVDSLAKEFMKQALPPRLTEQEKKLSVLGSSTNLLGDDL----VDFTARTKVRLIRR
D. melanogaster   IDEAVDQMAKKFQHEALPPIVLPSEEVRTVHGARSDADEQGNCVCDYKFNKKTSVRLLRA
H. vulgaris       IDEAGDELVLDHMEEFQPPYYSSDDVECSVFSKDGFWDGEVRRHHK--IELSTELRLVRP
H. sapiens        VDAVADQRAKDFIHDSLPPVLTDRERALSVYGLPIRWEAGEPVNVGAQLTTETEVHMLQD
M. musculus       VDAVADQRAKDFIHDSLPPVLTDRERALSVHGLPVRWEAGEPVNVGAQLTTETQVHMLQD
G. gallus         IDAAMDQRAKSFLHDCLPPVLTQSEKQLSVYGFPARWQDGGPRNVDIQITKDTEVRLLHH
D. rerio          VDAAVDQKAKDFLHDCLPPLLTAEEKAGSVYGAPARWGDSEALDVAVELKSQTRIKLVRA
                  ::   *. . .. .:  **     :   :* .                  :   * :::::

C. elegans        HTQRLLMESEDACFISHRINNSRLFEGRPEQIVEYPISGIDAYRVLSNSYPEWRTLYEIF
D. melanogaster   NILRLVTESDGSVRIYHHVDNGLDYCKYEPYFMEILPEEAKAVELLISAYPFYLTIDQL-
H. vulgaris       GIVRVIPA-DTELQVYYSVENSRQYKEVPLRVLRFSLEDADLLEYFLFSYPAYVVVENA-
H. sapiens        GIARLVGE-GGHLFLYYTVENSRVYHLEEPKCLEIYPQQADAMELLLGSYPEFVRVGDL-
M. musculus       GVARLVGE-GGRLFLYHTVENSRVYHLEEPKCLEIHPQQADAMELLLRSYPEFVRVGDL-
G. gallus         GVVRLCNE-EAGVMLYYTTENSRVYHKEEPKYLEIDPEYTDSIEFLLSSYPNHVSVDTL-
D. rerio          GAARLCSD-GDTVHLYYTTENSRVYHKEASKSFEMKTEHIDAMEFLIHSYPKFVSVASL-
                     *:          : :  :*.  :         ..   .   .   . :  :**     :

C. elegans        SLRETKTKSRKENLAAIQLLFQIGVLLVKN-----
D. melanogaster   -----PLESSARKIEVATALWEHGLLMTEKPFK--
H. vulgaris       -----PGDDIH-KVDVANRLYNFGILRSKVPLVHT
H. sapiens        -----PCDSVEDQLSLATTLYDKGLLLTKMPLALN
M. musculus       -----PCDSVEDQLSLATMLYDKGLLLTKTPLVPS
G. gallus         -----PCDALEDKISLATLLFEKGILTTKKPLVQV
D. rerio          -----PCETAEAKMSLAELLFEKGLIFTAEPLTAQ
                         .     ::     *:: *::
```
