# Supplementary material for: Phylogenetic and genomic analyses of the ribosomal oxygenases Riox1 (No66) and Riox2 (Mina53) provide new insights into their evolution
Source: BMC Evol Biol. 2018 Jun 19;18:96. doi: 10.1186/s12862-018-1215-0 (PMC6006756; doi:10.1186/s12862-018-1215-0)
Supplement: Supplementary file 12 — Clustal omega alignment of Riox2 protein sequences from H.vulgaris, zebrafish, chicken, mouse and human. The protein domains JmjC (red), dimerization (brown) and winged-helix (blue) are indicated based on the human sequence [16]. The prospective iron-binding motif HxD…H (green) and the 2OG C5-carboxylate-binding residue (K, purple) are conserved in all species. Crystal structure analysis of human RIOX2 with substrate Rpl27a identified the aa N101, Q136, Q139, N165 and S257 of human RIOX2 involved in Rpl27a peptide binding [16] (red). (PDF 90 kb) [file 12862_2018_1215_MOESM12_ESM.pdf]

Additional file 12: Figure S12

```
                                                                  JmjC domain
H. vulgaris    MVKRKGSSSDD-----------------VAKNVTKKVKKVSDVNEQKESKLNYTSCESVM
D. rerio       MPKISRRAARRSGQEERLQRVSSASSPATQRSGSPQEIEQEERLQRVSTLSSSGSPQEFF
G. gallus      MPKKRGKHAEM--GME----------------MQVQSKRAKVETDCSPSVMDFESPESLF
H. sapiens     MPKKAKPTGSG--KEE----------------GPAPCKQMKLEAAGGPSALNFDSPSSLF
M. musculus    MPKKVQPTGDE--NEE----------------ASVPCKRVKEELPETLSVLNFDSPSSFF
               * *     .                         . .        :  .  * ...:
                                                                           N
H. vulgaris    SSLLQPISVKTFFEEFWEKKPLYIKREN---SGYYGDLFSLSSMKEILAAHELEFETDVN
D. rerio       QSLIRPLDLQEFFQRFWERQPLVLHRSDAALAGYYGSLFPLSGLRRL-CARGLQYGTDIN
G. gallus      ASLISPIKPEVFFKEYWEEKPLLVQRNNPLLAAYYQSLFQLSDLKEL-CSQGLYYGRDIN
H. sapiens     ESLISPIKTETFFKEFWEQKPLLIQRDDPALATYYGSLFKLTDLKSL-CSRGMYYGRDVN
M. musculus    ESLISPIKVETFFKEFWEQKPLLIQRDDPVLAKYYQSLFSLSDLKRL-CKKGVYYGRDVN
                **: *:. : **:.:**.:** ::*.:   : ** .** *:.:: : . : : :  *:*
                                                 Q  Q
H. vulgaris    VCRYVDNEKELLNEDGCLTVDKFDKLMNDKHATFQLHQPQRYGTVLWQLMEKMETYFGCL
D. rerio       TCRCVRGQKRLLNRAGAVDFCLLERDFLEKKATIQFHQPQRFQDELWRIQERLECFFGCL
G. gallus      ICRCVNGKKKVLNKEGKVNYAQLKKDFDQKKATIQFHQPQRFKEELWKIQEKLECYFGSL
H. sapiens     VCRCVNGKKKVLNKDGKAHFLQLRKDFDQKRATIQFHQPQRFKDELWRIQEKLECYFGSL
M. musculus    VCRSISGKKKVLNKDGRAHFLQLRKDFDQKRATIQFHQPQRYKDELWRIQEKLECYFGSL
                ** : .:*.:**. *      : : : :*:**:*:*****:   **:: *::* :**.*
   N          H D            K
H. vulgaris    VGSNVYITPKESQGLAPHCDDVEVFILQLEGTKHWKLYKPMVELSRDYTQDLSQDSIGEP
D. rerio       VGSNVYITPAGAQGLPPHYDDVEVLILQLEGQKHWRLYEPTVPLAREYSLE-PEGRIGAP
G. gallus      VGSNVYITPQGSQGLPPHYDDVEVFILQLEGEKHWRLYKPTVHLAREYNVE-SEDRIGNP
H. sapiens     VGSNVYITPAGSQGLPPHYDDVEVFILQLEGEKHWRLYHPTVPLAREYSVE-AEERIGRP
M. musculus    VGSNVYMTPAGSQGLPPHYDDVEVFILQLEGTKHWRLYSPTVPLAHEYSVE-SEDRIGTP
               ******:**  :*** ** *****:****** ***:** * * *::*. :  :  ** *
                  H                S      dimerization domain
H. vulgaris    IMELTLEPGDLLYFPRGTIHQARS-VGESYSTHITLSTYQN-------------------
D. rerio       THDFILQAGDLLYFPRGTIHQADTPAGAGHSTHLTLSTYQNMCVCAVHNVTHTHTHTLQN
G. gallus      THEFVLKPGDLLYFPRGTIHQADTPPGIPYSTHVTISTYQN-------------------
H. sapiens     VHEFMLKPGDLLYFPRGTIHQADTPAGLAHSTHVTISTYQN-------------------
M. musculus    THDFLLKPGDLLYFPRGTIHQAETPSGLAYSIHLTISTYQN-------------------
                 :: *: ************** :   *   :* *:*:*****

H. vulgaris    --------------NTLGDFMSIAVSQAIESALENDVNFRRGLPINYLSYLGTAKNFSKY
D. rerio       VLVLHSFIVCVRACRSWGDLLLDLMPGCVFDRMKTDCELRTGLPRGLLTTPSISPAVSHQ
G. gallus      --------------NSWGDFLLDAIPGLVFSTAKDDVALRTSIPRKLLMQVDI-ADSTKK
H. sapiens     --------------NSWGDFLLDTISGLVFDTAKEDVELRTGIPRQLLLQVESTTVATRR
M. musculus    --------------NSWGDCLLDSISGFVFDIAKEDVALRSGMPRRMLLNVETPADVTRK
                             .: ** :   :   : .  : *  :* .:*   *         ::
                                                        winged helix domain
H. vulgaris    FDEDGKESKLSSNENNEKVKKFKDSVKKHLSKLIDHIDVNTAADMMSYDFMASRLPPFGH
D. rerio       ----------------------LSVFLQRLADVVDHQGQTLRSSSMRRDFISHRLPPFVQ
G. gallus      ----------------------LSSILRMLADRLENTG-ELRSSDMRKDFIMNRLPPCLG
H. sapiens     ----------------------LSGFLRTLADRLEGTK-ELLSSDMKKDFIMHRLPPYSA
M. musculus    ----------------------LSGFLRTLADQLEGRE-ELLSSDMKKDFVKHRLPPFFE
                                      . . : *:. ::       :. *  **:  ****

H. vulgaris    VVKEEQLN-EFKSPTLDSKIKLRYPEHVRPVYY-DQEETDEADNTVGDDDSEDEEEETVK
D. rerio       --DPQLLQPVGGAPALQDTVSLRFKDHLLLTVEPSPDHTD--------------------
G. gallus      C-DSDSLTPGGKVPKLDSKIRLQFRDHAVITVEPDQENSD--------------------
H. sapiens     GDGAELSTPGGKLPRLDSVVRLQFKDHIVLTVLPDQDQSD--------------------
M. musculus    GNGTETMDPGKQLPRLDNIIRLQFKDHIVLTVGPDKNPFD--------------------
                   :         * *:. : *:: :*   .    . :   *

H. vulgaris    KDEDEKNEEKDEKSPKSNKKSSDKKDTSMEGNEDEEDEDDDDASVHDDEPCIKIVHSLNN
D. rerio       ---------------------------------------------EATELLVYVLHSLRN
G. gallus      ---------------------------------------------EIRKEMVYVVHSLKN
H. sapiens     ---------------------------------------------EAQEKMVYIYHSLKN
M. musculus    ---------------------------------------------EAQQKVVYIYHSLKN
                                                            .  :  : : ***.*

H. vulgaris    NRETHMSGHDLS----------RDVFSLKLPIHFAQAITSVMNSNDFICVRDLPLDDDED
D. rerio       RRDTHMMMGASDED---EDDEESQVGGLRFPLSHLEALQQLLVS-DRVPVEDLQLQQ-ED
G. gallus      RRETHMMGTEDDDTGSEEGAAQQTPHGLRFPLSYLDALKQIWSG-STVSVKELKLTSAEE
H. sapiens     SRETHMMGNEEE----------TEFHGLRFPLSHLDALKQIWNS-PAISVKDLKLTTDEE
M. musculus    VRQMHMIGEEEE----------SEIFGLRFPLSHVDALKQIWCG-SPIRVKDLKLDTDEE
                *: **     .              .*::*: . :*: .:   .    : *.:* *   *:

H. vulgaris    KLQLATSLYSDDLIEIK-------
D. rerio       KLNLLLALWSEGLLRVTGALENHH
G. gallus      KENLALALWTECLIEVF-------
H. sapiens     KESLVLSLWTECLIQVV-------
M. musculus    KENLALSLWSESLIQVL-------
               * .*  :*::: *:.:
```
