# Supplementary material for: Phylogenetic and genomic analyses of the ribosomal oxygenases Riox1 (No66) and Riox2 (Mina53) provide new insights into their evolution
Source: BMC Evol Biol. 2018 Jun 19;18:96. doi: 10.1186/s12862-018-1215-0 (PMC6006756; doi:10.1186/s12862-018-1215-0)
Supplement: Supplementary file 13 — Clustal omega alignment of Rpl8 (A) and Rpl27a (B) protein sequences from C. intestinalis, H. vulgaris, C. elegans, A. melifera, fruit fly, zebrafish, X. laevis, chicken, mouse and human. The protein sequence corresponds to the aa 208–224 of human RPL8 (A) and aa 30–51 of human RPL27A (B). Both aa-stretches harbour the H-residues, which get hydroxylated by human RIOX1 (H216 in RPL8) and RIOX2 (H39 in RPL27A). Sequences highlighted in red are species where no Riox2 gene has been identified. (PDF 62 kb) [file 12862_2018_1215_MOESM13_ESM.pdf]

Additional file 13: Figure S13

A

Rpl8

```
                208      216     224
                 |        |       |
H.sapiens       EHPFGGGNHQHIGKPST

M.musculus      EHPFGGGNHQHIGKPST
G.gallus        EHPFGGGNHQHIGKPST
X.laevis        EHPFGGGNHQHIGKPST
D.rerio         EHPFGGGNHQHIGKPST
D.melanogaster  EHPHGGGNHQHIGKAST
A.mellifera     EHPHGGGNHQHIGKAST
C.elegans       EHPHGGGNHQHIGHPST
H.vulgaris      EHPHGGGNHQHIGFPST
C.intestinalis  EHPFGGGNHQHIGSPST
                *** *********  **
```

B

Rpl27a

```
                30       39          51
                 |        |           |
H.sapiens       GGRGNAGGLHHHRINFDKYHPG

M.musculus      GGRGNAGGMHHHRINFDKYHPG
G.gallus        GGRGNAGGMHHHRINFDKYHPG
X.laevis        GGRGNAGGMHHHRINFDKYHPG
D.rerio         GGRGNAGGLHHHRINFDKYHPG
D.melanogaster  GGRGNAGGMHHHRINFDKYHPG
A.mellifera     GGRGNAGGLHHHRINFDKYHPG
C.elegans       GGRGNAGGQHHHRINRDKYHPG
H.vulgaris      GGRGKAGGQHHHRILMDKYHPG
C.intestinalis  GGRGNAGGQHHHRINYDKYHPG
                **** *** *****  ******
```
